# Preparation of Human Milk Substitute Fat by Physical Blending and Its Quality Evaluation

**DOI:** 10.3390/foods15010081

**Published:** 2025-12-26

**Authors:** Xueming Jiang, Yuting Fu, Chunyi Song, Wendi Zhang, Jun Cao

**Affiliations:** Key Laboratory of Food Nutrition and Functional Food of Hainan Province, School of Food Science and Engineering, Hainan University, Haikou 570228, China; 18281565277@163.com (X.J.); 24110832000014@hainanu.edu.cn (Y.F.); scy23468@163.com (C.S.); 22210832000005@hainanu.edu.cn (W.Z.)

**Keywords:** human milk fat substitutes, preparation and quality evaluation, fatty acids, sn-2 fatty acids, sn-2 palmitoyl triacylglycerols, oxidation and flavor

## Abstract

Human milk is the benchmark for formulating infant formula, the latter serving as a substitute when breastfeeding is not possible. Nevertheless, the lipid composition and structure of commercially available infant formulas still differ from those of human milk fat. Accordingly, this paper employs a computational–experimental framework to optimize formulations of prepared lipid (PF). The quality of the optimized product was further validated by analyzing volatile organic compounds (VOCs), color, lipid oxidation indicators, and oxidative stability. The results show that a total of 43 fatty acids (FA) were detected in the base oil, and palmitic acid, oleic acid, and linoleic acid are the main types of FA. Through computer simulation, 6 of PF were obtained, which are superior to commercial products (SP) in the similarity score of the parsimonious model, and PF1 has the highest score (84.15). Multivariate statistical analysis indicates that PF may be more suitable for use in infant formula milk powder due to its lipid composition. Gas chromatography-ion mobility spectrometry was used to study the VOCs content of PF and SP, and a total of 35 VOCs were identified. It was found that alcohols and ketones accounted for the highest proportion in PF, while Nitriles, Aldehydes, and Esters were the most abundant in SP. In the comparison of the basic physical and chemical indices between PF and SP, the peroxide value and *p*-anisidine value of PF are lower, and the overall oxidation stability is stronger than that of SP. This study provides a reference for the preparation and multi-dimensional evaluation of human milk fat substitutes.

## 1. Introduction

Optimal infant growth and development depend on a balanced provision of macro- and micronutrients, among which lipids serve as one of the critical components. Yet, the lipid composition of conventional infant formula often remains suboptimal compared to the biological benchmark set by human milk fat (HMF). To bridge this nutritional gap, significant research is dedicated to developing human milk fat substitutes that more closely mimic the structure and function of HMF. The ultimate goal is to provide formula-fed infants with a lipid source that better supports their physiological and developmental requirements. Human milk is the most ideal food source for infants and young children, providing 45–50% of energy, essential fatty acids, and various vitamins and other nutrients [1,2]. Human milk contains 3–5% of lipids, among which as high as 98% exists in the form of triglycerides (TAG) [3]. The fatty acid composition in HMF mainly includes palmitic acid (26–35%), oleic acid (19–24%), and linoleic acid (10–18%) [4,5]. HMF contains a small amount of polyunsaturated fatty acids (PUFA), and although the content is small, it cannot be ignored. Adding docosahexaenoic acid (DHA) and arachidonic acid (ARA) to infant formula can improve vision and cognitive function [6]. The characteristic sn-2 esterification of saturated fatty acids (SFA), predominantly palmitic acid, which constitutes approximately 60–75% of this positional fraction in human milk TAG, reduces the formation of insoluble calcium soaps, thereby lowering the associated risks of calcium deficiency, constipation, and distress in infants and young children [7,8]. In HMF, 1,3-dioleoyl-2-palmitoyl (OPO) and 1-oleoyl-2-palmitoyl-3-linoleoyl (OPL) are the main TAG types, but their specific proportions vary due to factors such as regions, living habits, and the physical conditions of mothers [9]. For instance, in investigations of breast milk from various Chinese regions, including Zhengzhou, Harbin, and Wuhan, OPL was consistently found to be more abundant than OPO across lactation stages, reflecting regional dietary patterns characterized by high intake of linoleic acid-rich oils such as soybean oil [10,11]. In contrast, studies from Egypt, Spain, and Denmark reported OPO as the predominant triacylglycerol in human milk fat, which aligns with dietary habits featuring higher consumption of oleic acid-rich oils such as olive oil [10,12,13]. These regional and dietary influences underscore the importance of developing human milk fat substitutes that are tailored to local nutritional profiles and lactation physiology.

When breastfeeding with human milk is not feasible, infant formula is a good alternative. Infant formula manufacturers and researchers have always developed infant formula based on the characteristics of human milk, aiming to meet the nutritional needs of infants during their growth. The research and development of human milk fat substitutes is one of the key aspects in infant formula production, and it is prepared according to the components of HMF. The main preparation methods of human milk fat substitutes include physical blending and structured lipid preparation methods [9]. Physical blending prepares human milk fat substitutes by simply mixing different animal fats, vegetable oils, or microbial oils. It is easy to operate and generates no by-products, so it is commonly used in large-scale commercial production. For instance, in the early days, bovine butterfat was used as a human milk fat substitute in infant foods. Later, it evolved into a mixture of multiple vegetable oils and animal fats, and PUFA, such as DHA and ARA, were also added. However, this method cannot change the distribution of FA on TAG, and thus cannot fully meet the nutritional needs of infants [14,15]. Structured lipid preparation primarily involves three enzymatic approaches: enzymatic acidolysis, enzymatic transesterification, and enzymatic esterification. These methods have been progressively refined with advancements in immobilized lipase technology and process design. Enzymatic acidolysis offers straightforward operation and facilitates product separation. Historically constrained by acyl migration, which compromised product purity, the process has been improved through optimized reaction conditions—such as maintaining temperatures between 40 and 60 °C and limiting reaction time to 3–5 h—and the use of sn-1,3 regioselective immobilized lipases (Lipozyme RM IM, Lipase UM1). These strategies have effectively minimized acyl migration, enabling retention rates of sn-2 palmitic acid exceeding 70% in human milk fat substitutes production [16,17]. Enzymatic transesterification is characterized by high selectivity and minimal by-product formation. Recent developments in carrier-modified immobilized lipases (CSL@OMS-C_8_, SOM-MIX@PDMS@RML) have significantly enhanced reaction specificity and synthesis efficiency. Coupled with streamlined purification techniques, such as molecular distillation and dry fractionation, separation challenges have been overcome, yielding target lipids at rates above 80% [18,19]. Enzymatic esterification allows direct utilization of free fatty acids and glycerol from by-products. While previously limited by operational complexity and high costs, these constraints have been mitigated through solvent-free reaction systems and multi-step cascade processes that simplify operations. Moreover, the strong reusability of immobilized lipases—for instance, Lipozyme TL IM retaining 91.5% activity after 15 cycles—has reduced production costs, making this method increasingly viable for industrial applications [20,21].

To deeply explore the similarity degree between human milk fat substitutes and HMF, researchers have constructed multiple evaluation models [22]. Innovatively put forward an evaluation model based on the optimal distance method. This model comprehensively evaluates the similarity between human milk fat substitutes and HMF from four dimensions, namely TAG, FA, sn-2FA, and TAG with special structures. Evaluated the similarity of lipid compositions between infant formula and human milk by optimizing the Bray–Curtis similarity index, which covers many key aspects such as fat content, fatty acids, TAG positional isomers, and phospholipid content [23]. Indexes such as chrominance, iodine value, and saponification value can effectively characterize the sensory quality, chemical properties, and oxidation degree of human milk fat substitutes. Moreover, these indices cooperate with each other and complement each other, jointly building a relatively complete quality evaluation system [24,25].

In this study, a variety of animal and vegetable oils, commercially available human milk fat substitutes, and human milk were used as materials. Taking HMF as the object, a simulation model was constructed with the help of MATLAB optimization instructions, and multiple evaluation models were employed for assessment. The aim was to accurately obtain the optimal formula combination of human milk fat substitutes, analyze its quality differences from multiple dimensions, such as the characteristics of volatile organic compounds, color characterization, and basic oil oxidation indicators, and thus achieve the quality evaluation of human milk fat substitutes. The aim of this study is to investigate the formulation of human milk fat substitutes and analyze their quality. The work provides a theoretical basis and data support for the rational selection of oils in infant formula, thereby offering references for optimizing human milk fat substitutes to better meet the nutritional requirements of infant growth and development.

## 2. Materials and Methods

### 2.1. Materials and Reagents

Analytical reagents such as methyl acetate, sodium methoxide, anhydrous sodium sulfate, sodium cholate, calcium chloride, and formic acid were purchased from Sinopharm Chemical Reagent Co., Ltd. (Shanghai, China) Acetonitrile and isopropanol were of chromatographic purity and were purchased from Sigma-Aldrich. OPO (Yuanye, Shanghai, China); OPL (Zzbio, Shanghai, China); GLC #463 (NuChek Prep, Elysian, MN, USA). Fourteen of oils (corn oil, flaxseed oil, soybean oil, low erucic rapeseed oil, sunflower oil, coconut oil, palm oil, walnut oil, rice bran oil, peanut oil, perilla seed oil, camellia oil, butterfat, and lard) and three of fish (tilapia, golden pompano, and basa catfish) were purchased from supermarkets in Hainan, China. Two of the commercially available human milk fat substitutes were obtained. Reference human milk samples were sourced from a laboratory repository, originating from human mature milk donations (three months postpartum) provided by 52 volunteers (aged 25–35 years) in Hainan, China. All repository samples were preserved in sterile tubes under controlled storage conditions at −80 °C.

### 2.2. Sample Preparation

Extraction and fractionation of fish oil: Take 50 g of fish meat samples, mash them, and place them together with 5 mg of butylated hydroxytoluene in a 500 mL beaker. Add 150 mL of dichloromethane: methanol (1/2, *v*/*v*) and shake. Then add 50 mL of ultrapure water and centrifuge at 5000 rpm for 15 min. Take the lower dichloromethane layer, and add another 50 mL of dichloromethane to the remaining liquid for repeated extraction. Combine the dichloromethane phases in a rotary evaporation flask and rotary evaporate at 65 °C to obtain the total lipids [26]. At −12 °C, conduct fractionation according to the solid–liquid ratio (fish oil:isopropanol = 1:4) for 4 h [21]. Then centrifuge at 10,000 rpm for 10 min to obtain liquid oil and solid fat. The isopropanol was dried with nitrogen gas.

Extraction of human milk lipids: Take 1 mL of human milk and mix it thoroughly with chloroform/methanol (1:1). Then, centrifuge the mixture at 5000 r/min for 10 min. Take out the chloroform layer, blow it dry with nitrogen, and store it at −4 °C for later use [27].

### 2.3. Fatty Acid Analysis by Gas Chromatography-Flame Ionization Detector

The methylation method refers to our previous work [28], using an Agilent 7890A gas chromatograph-flame (Agilent Technologies Inc., Santa Clara, CA, USA) ionization detector and a CP-Sil 88 capillary column (100 m × 0.25 mm × 0.2 μm). The column temperature was set at 45 °C, then increased to 175 °C at a rate of 13 °C/min and held for 4 min, and further increased to 215 °C at a rate of 4 °C/min and held for 35 min.

Take about 10 mg of oil and place it in a centrifuge tube. Add 10 mL of Tris-HCl solution (1 mol/L, pH 7.6), 2.5 mL of cholate solution (0.05%, *w*/*v*), 1 mL of calcium chloride solution (2.2%, *w*/*v*), and 10 mg of porcine pancreatic lipase. Shake at 37 °C for 10 min, terminate the reaction with ether, centrifuge, and take the supernatant. Blow dry with nitrogen and redissolve with chloroform. Spot on a thin-layer plate, air-dry, and develop with a developing solvent (n-hexane: ether: formic acid, 50:50:1, *v*/*v*/*v*). Take it out, air-dry, and develop color in an iodine tank for 30 s. Scrape off the monoglyceride and extract it with chloroform, centrifuge, and combine the supernatant. Blow dry with nitrogen to obtain monoglyceride, dissolve it in boron trifluoride-methanol solution (14% in methanol), shake in a water bath (90 °C, 10 min), then add 2 mL of distilled water and 2 mL of n-hexane to terminate the reaction. Centrifuge and take the liquid, pass it through a column, and blow dry with nitrogen to obtain fatty acid methyl Esters for GC detection. Identify fatty acids qualitatively with GLC #463 chromatogram and quantify them by the area normalization method [29,30]. The relative content of palmitic acid at the sn-2 position was calculated according to the formula:

(1) $$W_{1}\%\;=\;\mathrm{Wp}1/(3\times \mathrm{Wp}2)\times 100\%$$

W_1_ represents the relative content of palmitic acid at the sn-2 position (in percentage); Wp1 represents the percentage content of palmitic acid at the sn-2 position in the total fatty acids; Wp2 represents the percentage content of all palmitic acid in the lipids in the total fatty acids.

### 2.4. Analysis of Triglyceride Composition

OPO and OPL in lard, fish oil, HMF, SP, and PF were determined using HPLC-ELSD. Take about 10 mg of lipids, add 1 mL of n-hexane, and shake well. Filter through a 0.22 μm organic filter membrane into the sample vial. Use HPLC-ELSD (1260 Infinity Ⅱ, Agilent Technologies Inc., Santa Clara, CA, USA) to measure OPO and OPL. The chromatographic column is ZORBAX Eclipse Plus C18 (4.6 mm × 250 mm, 5 μm), the injection volume is 10 μL, the ELSD temperature is 55 °C, the nitrogen flow rate is 1.8 L/min, the gain is 1, the mobile phase A is acetonitrile, and B is isopropanol. The elution program is as follows: from 0 to 30 min, 30–40% B; from 30 to 70 min, 40–45% B; from 70 to 100 min, 45% B; from 100 to 105 min, 45–30% B. The flow rate is 0.8 mL/min [3]. Draw standard curves with the peak area and the concentration of the standard solution (for OPO: y = 2476.4x^1.784^, R^2^ = 0.9056; for OPL: y = 2635.5x^1.7975^, R^2^ = 0.9511). Qualitative and quantitative analyses are carried out by the external standard method. Notes, it was emphasized that OPO and OPL identified in this study might contain other regioisomers such as OOP/POO and OLP/POL, because HPLC-ELSD could not separate isomers.

### 2.5. Preparation and Evaluation of PF and SP

The formulation of PF was computationally optimized using MATLAB; the optimization procedure was structured as follows:

[x, fval] = linprog (f, A, b, Aeq, beq, lb, ub)(2)

x: the optimal value;

fval: the function value returned by the objective function when the optimization variable x obtains the optimal solution;

f: the coefficient matrix of the optimization variable x. In this study, since the cost is not considered, the coefficients can be set as any number greater than 0;

A: the coefficient matrix of the optimization variable x in the inequality constraint function A * x ≤ b;

b: the matrix of the constraint conditions in the inequality constraint function A * x ≤ b;

Aeq: the coefficient matrix of the optimization variable x in the equality constraint function Aeq * x = beq;

beq: the matrix of the constraint conditions in the equality constraint function Aeq * x = beq;

lb and ub are the lower and upper bounds of the optimization variable x, respectively.

Evaluation of the similarity between PF, SP, and HMF:

(3) $$G_{FA/sn\text{-}2FA/TAG}=\sum_{i=1}^{n}\left[(1-\frac{\left|b_{i}-a_{i}\right|}{a_{i}})\times 100\right]/n$$

(4) $$G=(G_{FA}+G_{sn\text{-}2FA}+G_{TAG})/3$$

In the formula: G_FA/sn-2FA/TAG_ represents the scores for FA, sn-2FA, and TAG, respectively; ai is the upper or lower limit of the content of the corresponding FA, sn-2FA, or TAG in HMF; b_i_ is the content of this indicator in human milk fat substitutes. If b_i_ is higher than a_max_, a_i_ takes a_max_; if b_i_ is lower than a_min_, b_i_ takes a_min_; when b_i_ is between the upper and lower limits of a_i_, the similarity is recorded as 100; n is the number of indicators under investigation; G is the total score.

### 2.6. Analysis of the Aromatic Compounds

Volatile organic compounds (VOCs) in lipid samples were analyzed under mild conditions using headspace sampling coupled with gas chromatography–ion mobility spectrometry (GC-IMS). Approximately 3 g of lipids was placed into a 20 mL headspace vial, incubated at 60 °C for 10 min under agitation (250 rpm), and 500 μL of the resulting headspace gas was then injected in split mode (split ratio 1:1) into the system. GC separation was carried out on an HP-5 non-polar capillary column (30 m × 0.32 mm, 0.25 μm) with a constant flow rate of 1.0 mL/min, using an injector temperature of 200 °C and the following oven program: 45 °C held for 12 min, increased to 130 °C at 3 °C/min, then to 180 °C at 8 °C/min and held for 2 min, yielding a total cycle time of 60 min including a 2 min post-run. IMS detection was performed at 45 °C with a 5.3 cm drift tube and high-purity nitrogen (≥99.999%) as drift gas at 150 mL/min, while the IMS detector was also maintained at 45 °C [31,32,33].

### 2.7. Basic Physicochemical Indexes of PF and SP

Chrominance: After the lipids are melted, uniformly place them in an LED photography studio with the same light intensity and measure them using a Minolta CR-400 chromometer (Konica Minolta Sensing, Osaka, Japan).

Iodine value, Saponification value, Acid value, Peroxide value, *p*-anisidine value and Carbonyl value are determined, respectively, according to Chinese national standards GB/T 5532-2022, GB/T 5534-2024, GB/T 5534-2008, GB 5009.229-2016, GB 5009.227-2023, GB/T 24304-2024 and GB 5009.230-2016 [34,35,36,37,38,39,40].

Oxidation stability test: Conduct the test using an 892 Professional Rancimat (Metrohm, Herisau, Switzerland) under the conditions of 150 °C and a gas flow rate of 10 L/h. Take about 3 g of lipids and 80 mL of deionized water. When the electrical conductivity increases rapidly, it indicates that the endpoint of the experiment has been reached.

### 2.8. Statistical Analysis

The experimental results are expressed as mean ± standard deviation, and all experiments are repeated at least three times. Excel 2021 is used for preprocessing the experimental data; SPSS 26.0 software is used for statistical analysis. One-way analysis of variance and Duncan’s multiple comparison analysis are adopted for significance testing, and a *p* < 0.05 indicates a significant difference. MATLAB R2023a is used for data simulation; SIMCA 14.1 and Origin 2021 are used for graph plotting.

## 3. Results and Discussion

### 3.1. Lipid Composition of Base Oils

#### 3.1.1. Fatty Acid Profiles of Base Oils

**Figure 1:**
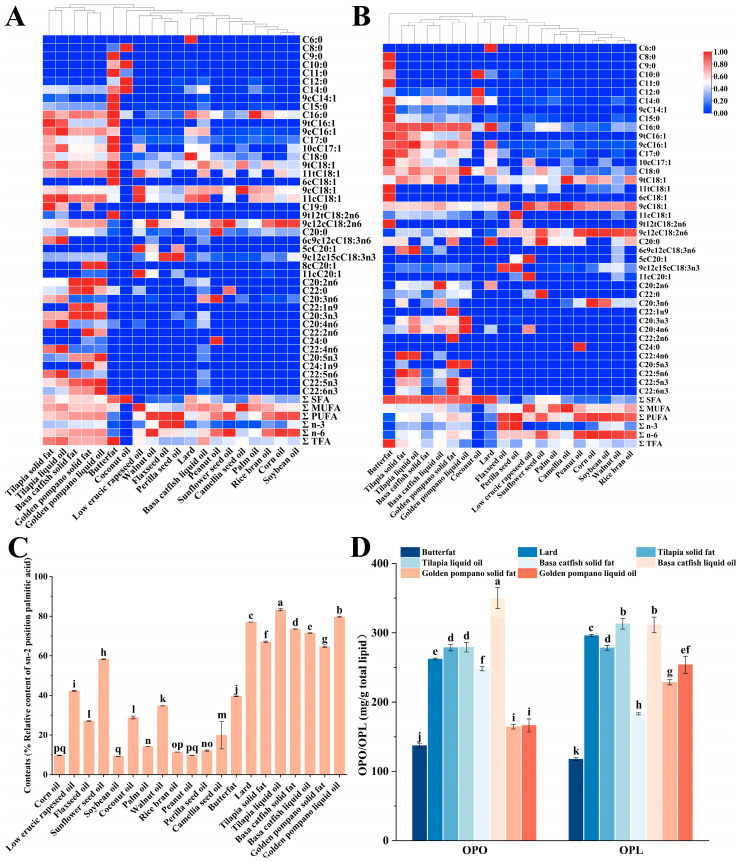
Visualization of the composition of total fatty acids (**A**) and sn-2 fatty acids (**B**) achieved by heat map analysis. Relative content of palmitic acid at the sn-2 position in different raw material oils (**C**), comparison of the contents of OPO and OPL in animal oils (**D**). Each colored square on the graph represents the corresponding peak area value of different lipid categories in different base oils. Red and blue represent high abundance and low abundance, respectively. In a bar chart, different lowercase letters represent significant differences (*p* < 0.05).

Clarifying the fatty acid (FA) composition of the selected base oils provides essential data for modeling prepared lipids to develop human milk fat substitutes. The quantitative profiles of major fatty acid classes (SFA, MUFA, and PUFA) and key individual FA varied considerably among the different oil sources. For instance, perilla seed oil was rich in α-linolenic acid (C18:3n3), accounting for 58% of total fatty acids, while camellia oil exhibited high oleic acid (9cC18:1) content, reaching 77.68% of total fatty acids. These compositional differences highlight the distinct nutritional characteristics of each oil, which should be considered when designing lipid blends intended to mimic HMF (Table S1). In most of the basal oils, their major FAs were palmitic acid (C16:0), oleic acid (9cC18:1), and linoleic acid (9c12cC18:2n6), which accounted for 4.36–36.84%, 6.67–77.68%, and 7.70–60.17% (Figure 1A, Table S1). In addition, coconut oil has a high saturated fatty acid (SFA) content of 91.79%, which is much higher than other animal and vegetable oils. Its SFA were mainly composed of lauric acid (C12:0) and myristic acid (C14:0), which accounted for 50.63% and 21.93%, respectively. Studies have shown that lauric acid and myristic acid are considered natural antimicrobial agents [41,42]. However, it is worth noting that the total amount of lauric acid and myristic acid should be no more than 20% of the total FA as stipulated in GB10765-2021 [43], so it is necessary to consider controlling the proportion of coconut oil when considering the preparation of PF. α-Linolenic acid (ALA, 9c12c15cC18:3n3) was found in high content in flaxseed oil and perilla seed oil, as 52.47% and 58.00%, respectively (Table S1), 52.47% and 58.00%, respectively (Table S1). ALA is one of the essential unsaturated fatty acids, which can be synthesized into EPA in the body by Δ6 desaturase, a chain-lengthening enzyme, and Δ5 desaturase, and then converted into DHA by β-oxidation. However, the efficiency of this series of enzymatic reactions is relatively low in humans, particularly the final step leading to DHA production, which is notably limited [44]. In addition, fish oil contains special polyunsaturated fatty acids, especially EPA and DHA, which contain 0.06–0.33% and 0.09–1.44%, respectively. EPA and DHA play a key role in the neurological, visual, and immune development of infants and children [45,46]. The composition of FA and its esterification position in triglycerides (TAG) are closely related to the bioavailability and physiological characteristics of TAG [47]. During the detection and analysis of base oils, a total of 40 sn-2FA are discovered (Figure 1B). The content of sn-2 C16:0 in animal fats ranged from 49.84% to 66.87%, significantly higher than that in vegetable oils (2.09% to 15.47%). Similarly, sn-2 C18:0 levels in animal fats (9.04–17.10%) exceeded those in most vegetable oils (1.54–9.42%), indicating a higher proportion of sn-2 LCSFA in animal fats. However, this structural advantage is accompanied by other compositional traits less favorable for human milk fat substitutes: animal fats exhibited substantially higher total SFA content (58.50–81.42% vs. a wide range of 5.75–92.95% in vegetable oils, the latter being skewed by exceptionally high SFA in coconut oil) and contained measurable levels of ΣTFA (0.18–1.98%). In contrast, most vegetable oils had negligible or lower ΣTFA (0.42–0.74%, except perilla seed oil at 0.35%). Furthermore, the ΣPUFA content was significantly lower in animal fats (4.12–21.86%) compared to vegetable oils (1.71–76.11%), which is a critical nutritional consideration for human milk fat substitutes. Regarding MUFA, the sn-2 C18:1n-9 content in most vegetable oils (23.29–79.02%) was substantially higher than in animal fats (8.43–22.05%), with exceptions such as coconut and perilla seed oils. Similarly, sn-2 C18:2n-6 levels were markedly higher in vegetable oils (18.57–66.32%, e.g., 66.32% in corn oil) than in animal fats (2.04–16.22%). Certain vegetable oils, such as flaxseed and perilla seed oils, were also rich sources of sn-2 C18:3n-3 (55.61%), whereas this fatty acid was minimal or undetectable in most animal fats. In summary, while animal fats provide a high sn-2 LCSFA content desirable for structural mimicry, their high SFA, presence of TFA, and low PUFA levels limit their standalone use in human milk fat substitutes. Conversely, vegetable oils offer higher sn-2 unsaturation and more favorable overall fatty acid profiles but are deficient in sn-2 LCSFA. These complementary profiles underscore the necessity of rationally blending animal and vegetable oils to achieve an optimal balance for human milk fat substitutes development (Figure 1B, Table S2).

#### 3.1.2. Comparison of TAG Containing Palmitic Acid at the sn-2 Position in Base Oils

Calculated according to Formula (1). If it was greater than 33.3%, it could be considered that the FA was mainly distributed at the sn-2 position of TAG; otherwise, it was considered to be mainly distributed at the sn-1,3 positions of TAG [48]. As shown in Figure 1C, the values of palmitic acid in different base oils ranged from 9.18 to 83.24%. For vegetable oils, only the values of low erucic rapeseed oil, sunflower seed oil, and walnut oil were reached. Based on this, it could be judged that palmitic acid in these three vegetable oils was distributed at the sn-2 position. In contrast, for animal oils, their values were all higher than 33.3%. Thus, it could be inferred that the palmitic acid of most vegetable oils might tend to be distributed at the sn-1,3 positions of triglycerides. The previous discussion had preliminarily proved that animal oils had the potential to be a source of sn-2 palmitic acid. Therefore, it was necessary to further compare the contents of OPO and OPL in 8 animal oils. As shown in Figure 1D, the contents of OPO and OPL in 8 animal oils were 137.06 mg/g total lipid, 350.01 mg/g total lipid, 117.60 mg/g total lipid, and 313.08 mg/g total lipid, respectively. Among them, the OPO content of basa catfish liquid oil was as high as 350.01 mg/g, which was significantly higher than that of the other 7 of animal oils; there was no significant difference in the OPL content between basa catfish liquid oil (311.49 mg/g total lipid) and golden pompano liquid oil (313.08 mg/g total lipid). However, compared with the other 6 of animal oils, the OPL contents of these two were significantly higher. Since HMF had a unique FA composition distribution and TAG structure, which was crucial for infants’ nutrient absorption and healthy growth [49]. Combined with the research results in Section 3.1.1, it showed that relying solely on vegetable oils or animal oils might have limitations when simulating HMF. Therefore, in the research and practice of simulating HMF, it was necessary to comprehensively consider the characteristics of vegetable oils and animal oils, and explore more reasonable oil combinations or modification methods to better meet the nutritional needs of infants.

### 3.2. Preparation and Similarity Evaluation of PF and SP

#### 3.2.1. Computer-Aided Formulation and Scoring Model Evaluation of SP

With the constraints of FA, sn-2FA, and OPO/OPL with more than 1% in HMF, we performed computer simulations using MATLAB software. Table S3 lists the details of the resulting outputs for the six PFs. In order to investigate the similarity between PF1-6, SP1-2, and HMF in depth, we used a resolving model for similarity scoring. The results show that PF1-6 scored higher than SP1 and SP2 in both G_FA_ and G_sn-2FA_ dimensions, and PF3 scored lower than SP1 and SP2 in the G_TAG_ scoring dimension. Overall, PF1 scored the highest score of 84.15 points. The total scores of all PF were higher than those of SP, indicating that they all have the potential to be added to the infant formula and contribute to improving the similarity of the product to human milk (Table 1).

**Figure 2:**
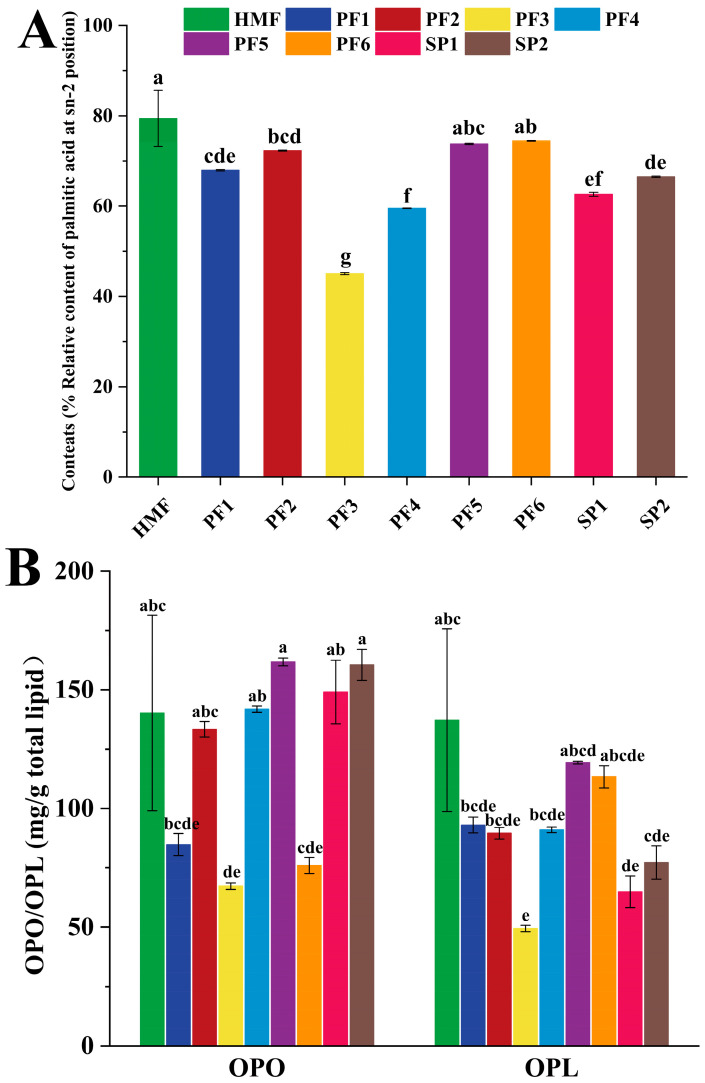
Relative content of palmitic acid at the sn-2 position (**A**), comparison of OPO and OPL contents (**B**) in human milk fat (HMF), six formulated lipids (PF 1-6), and two commercial products (SP 1-2). In a bar chart, different lowercase letters represent significant differences (*p* < 0.05).

As shown in Figure 2A,B, the relative content of sn-2 palmitic acid and the levels of OPO/OPL in samples PF1-6, SP1-2, and HMF are presented. The relative content of sn-2 palmitic acid in HMF was 79.41%, while PF5 and PF6 exhibited values of 73.77% and 74.45%, respectively. Statistical analysis indicated no significant difference in the relative sn-2 palmitic acid content among HMF, PF5, and PF6. In terms of OPO content, there was no significant difference in the OPO content of PF2, PF4, PF5, SP1, and SP2 compared with that of HMF in the PF1-6 and SP1-2 systems, with PF5 having the highest OPO content of 161.76 mg/g. In addition, except for PF3 and SP1, the rest of the PF and SP had no significant differences from HMF in terms of OPL content. In addition, except for PF3 and SP1, the other PFs and SPs did not differ significantly from HMF in OPL content. In general, the similarity of PF1-6 and SP1-2 could be evaluated objectively according to the unified rating system. Nevertheless, further in-depth evaluation is necessary to verify more accurately whether PF is closer to HMF in terms of composition and content of FA, sn-2FA, and OPO/OPL.

#### 3.2.2. Multivariate Statistical Analysis of PF, SP, and HMF

**Figure 3:**
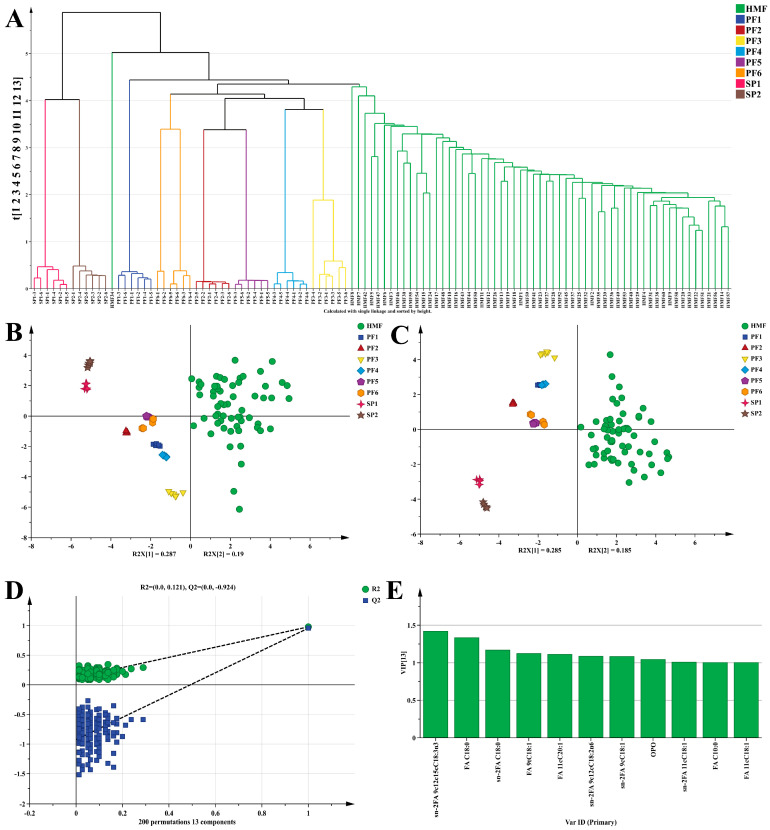
Lipid composition of PF and SP: Hierarchical cluster analysis (HCA) (**A**), PCA score plot (**B**), Two-dimensional score of PLS-DA model (**C**), and permutation plot of 200 cross-validations of PLS-DA model (**D**), VIP plot of the contribution of each variable to sample classification (**E**). VIP > 1, *p* < 0.01.

The FA, sn-2FA, and TAG of PF1-6, SP1-2, and HMF were again subjected to multivariate statistical analyses, which provided a deeper understanding of their basic characteristics. To accurately evaluate and identify the similarity between PF1-6, SP1-2, and HMF, three methods were adopted in this study. Firstly, hierarchical cluster analysis (HCA) was carried out based on the lipid information of the two. As can be clearly observed from Figure 3A, different samples show a very significant clustering effect. Except for a few HMF samples that were misclassified, the vast majority of PF1-6, SP1-2, and HMF samples were correctly classified. Specifically, there were distinct differences among PF1-6, SP1-2, and HMF. Compared with SP, PF was obviously more similar to HMF, and this result provides additional support for the earlier conclusion. Overall, HCA preliminarily confirmed that there was a certain similarity between PF1-6, SP1-2, and HMF. Then, an unsupervised PCA model was constructed to continuously explore the similarity between different PF1-6, SP1-2, and HMF. In the PCA plot, the closer the within-group distance was, the better the repeatability within the sample was; the farther the between-group distance was, the more significant the difference between the samples was. In the unsupervised PCA score plot Figure 3B, the explained variances PC1 and PC2 of the PCA model reached 28.7% and 19.0%, respectively, and the cumulative explained variance (R^2^Xcum) was 78.8%. This fully indicated that the constructed model was reasonable and could also demonstrate the characteristic that PF was closer to HMF. It was worth noting that although there was a certain degree of overlap between PF5 and PF6, the samples in Figure 3B generally tend to cluster according to different categories. Particularly noteworthy was that the distribution of HMF in the PCA plot was relatively scattered. Since PCA belonged to an unsupervised algorithm, HMF samples not only differed at the individual level but were also easily affected by various factors such as diet and living habits [50]. Therefore, the above factors were very likely to cause the within-group differences to become significant. If the within-group differences were large enough, they would inevitably affect the between-group differences, resulting in overlaps among different PF. In view of this, a supervised PLS-DA model was constructed to eliminate irrelevant factors such as within-group variance, effectively enlarge the between-group differences, and thus fully demonstrate the discriminant and predictive abilities of the model. As shown in Figure 3C, the classification trend of the PLS-DA model was consistent with that of the PCA model, with R^2^Xcum > 0.931, R^2^Ycum > 0.731, and Q^2^cum > 0.648. In the supervised mode, the differences between different PF1-6, SP1-2, and HMF became more significant, and the within-group differences in HMF decreased correspondingly. However, it should be noted that there was still a slight overlap between PF5 and PF6, which might be due to the presence of multiple base oils with the same source in their formulation compositions (Table S3). Fortunately, it was also firmly confirmed under this model that PF was closer to HMF compared with SP. The PLS-DA model was validated by a permutation test with 200 iterations Figure 3D, and the results were R^2^ = (0.0, 0.121) and Q^2^ = (0.0, −0.924). Obviously, the R^2^ and Q^2^ values after permutation on the left side were both lower than the initial points on the right side, which strongly indicated that the PLS-DA model did not have overfitting and the model construction was reasonable. Finally, to screen out the markers for distinguishing the three types of oils, namely SP, PF, and HMF, screening conditions (VIP > 1, *p* < 0.05) were set through VIP scores (Figure 3E). Then, 11 substances, including sn-2FA 9c12c15cC18:3n3, FA C18:0, sn-2FA C18:0, FA 9tC18:1, FA 11cC20:1, sn-2FA 9c12cC18:2n6, sn-2FA 9cC18:1, OPO, sn-2FA 11cC18:1, FA C10:0, and FA 11cC18:1, were obtained, and their contributions to distinguishing the samples were quite significant. In conclusion, these findings indicate that preparing PF via MATLAB-based computer simulation is feasible and suggest that this method may provide a useful reference for further research and practical applications.

### 3.3. Quality Evaluation of PF and SP

#### 3.3.1. Characterization of VOCs

**Figure 4:**
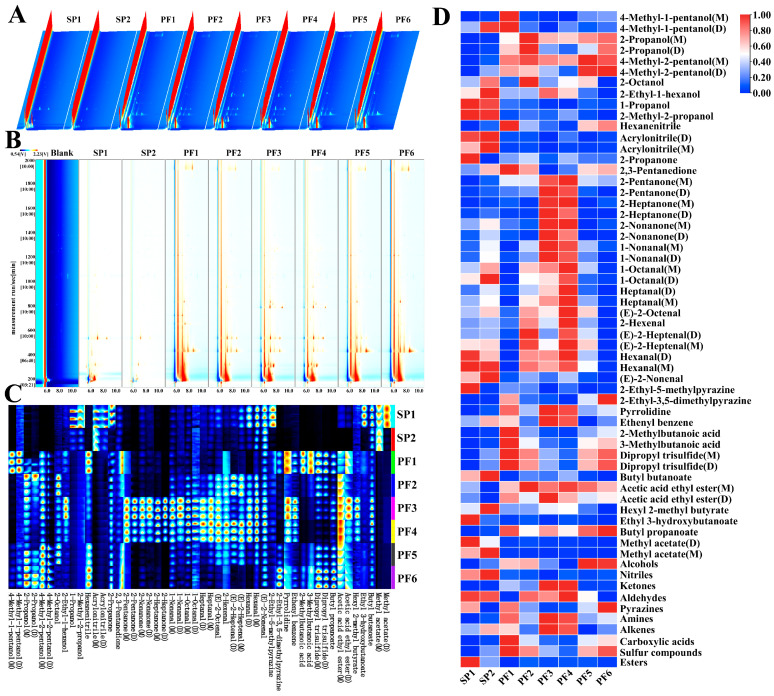
GC-IMS spectral analysis of volatile components in PF and SP: 3D topographic map (**A**); 2D spectrogram (**B**); fingerprint atlas (**C**), and heatmap visualization of volatile compounds (**D**).

He types, and content differences in volatile organic compounds (VOCs) had a very significant impact on the overall aroma characteristics of PF1-6 and SP1-2. Gas chromatography-ion mobility spectrometry (GC-IMS) was used to characterize the VOCs in different types of PF1-6 and SP1-2. Figure 4 shows the 3D spectrogram, 2D spectrogram, fingerprint atlas, and visual heatmap.

Figure 4A is the three-dimensional spectrogram of GC-IMS for PF1-6 and SP1-2. The three coordinate axes showed the drift time (horizontal axis), the retention time (vertical axis), and the signal peak intensity (vertical axis), respectively. It could be noticed that there were differences in the types and contents of VOCs among the PF1-6 and SP1-2. For the convenience of observation, the top view was further selected for comparison. The spectrum of Blank was chosen as the reference standard, and the normalized ion peak was obtained. The components matching its concentration are displayed in white, those with higher concentrations are shown in red, and those with lower concentrations are shown in blue (Figure 4B) [51,52]. It could be seen from the figure that most of the signals in the human milk fat substitutes samples appeared within the retention time range of 150–2000 s and the relative drift time range of 5–12 ms. Qualitative analysis was conducted with the help of the spectral library, and substances with significant concentration differences in this range were identified, mainly including alcohols, ketones, Aldehydes, sulfur compounds, and Esters. The dense gathering of highlights within the range of 200–600 s indicated that the abundances of Alcohols and Ketones were very high.

A total of 35 common VOCs were identified in the PF1-6 and SP1-2 samples. These compounds were classified into 10 categories, mainly including 7 of Alcohols, 2 of Nitriles, 5 of Ketones, 8 of Aldehydes, 2 of Pyrazines, 1 kind of Amines, 1 kind of Alkenes, 2 of Carboxylic acids, 1 kind of Sulfur compounds, and 6 of Esters (Table S4). The VOCs in PF1-6 and SP1-2 were qualitatively analyzed using the IMS database built into the software, and the fingerprint atlas was drawn using the “Gallery of evaluation area” plugin. The results are shown in Figure 4C. The horizontal axis represented the detected volatile substances, and the vertical axis represented the names of the corresponding samples. Each row represented the identified signal peaks within the corresponding sample, and each column represented the signal peaks of the same VOCs among different human milk fat substitutes. According to the research by Chen and Lin [53,54], when the ion signal of a compound could be clearly seen in a single sample, while the ion signal of this substance could not be observed or was extremely weak in other samples, it was generally assumed that this substance had a large difference among different samples. For example, the signal intensities of 1-Propanol, 2-Methyl-2-propanol, Acrylonitrile, Ethyl 3-hydroxybutanoate (D), and Methyl acetate (D) in SP1 were brighter than those in the other, indicating that their contents were higher. The visual heatmap was drawn according to the relative contents. For the convenience of comparison, the horizontal axis was standardized and normalized. The monomers and dimers of the same substance were represented by M and D, respectively. As shown in Figure 4D, the contents of Ketones in PF3 and PF4 were relatively high, such as 2-Pentanone (Fruit, Pungent), 2-Heptanone (Blue Cheese, Spice and Fruit, etc.), and 2-Nonanone (Fragrant, Fruit and Hot Milk, etc.). It was worth mentioning that PF4 also contained relatively high contents of Aldehydes, including 1-Nonanal (Fishy, Fat and Floral, etc.), 1-Octanal (Citrus, Fat and Green, etc.), heptanal (Citrus, Fat and Green, etc.), (E)-2-Octenal (Dandelion, Fat and Fruit, etc.), 2-Hexenal, (E)-2-Heptenal (Apple, Fat and Fresh) (Table S4). It should be noted that PF contained significantly higher isopropanol (Floral) than SP, which was most likely caused by the failure to completely remove 2-Propanol during the fish oil fractionation process. Through the analysis of the VOCs of PF1-6 and SP1-2, not only could the differences in their aroma characteristics be deeply understood, but also theoretical bases could be provided for the quality control and flavor optimization of products. Further research could be conducted on its formation mechanism and regulation methods, so as to regulate the flavor quality of PF more precisely. In addition, by combining with other analysis techniques, such as sensory evaluation, the flavor characteristics of PF could be evaluated more comprehensively, providing a more reliable basis for the research, development, and application of infant formula.

#### 3.3.2. Basic Physicochemical Indexes and Oxidation Stability

**Figure 5:**
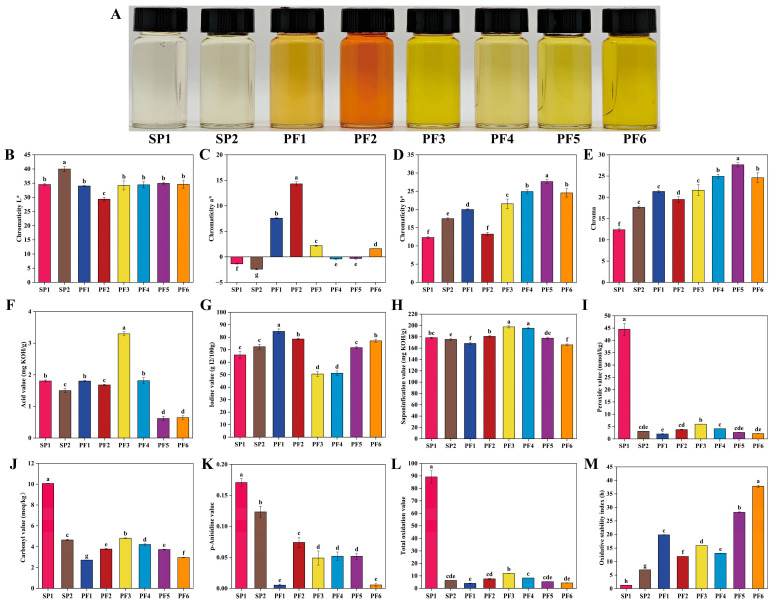
Comparison of physicochemical indexes for PF and SP: Physical display diagram (**A**), chromaticity L* (**B**), chromaticity a* (**C**), chromaticity b* (**D**), chroma (**E**), acid value (**F**), iodine value (**G**), saponification value (**H**), peroxide value (**I**), carbonyl value (**J**), *p*-anisidine (**K**), total oxidation value (**L**), oxidation induction period (**M**). In a bar chart, different lowercase letters represent significant differences (*p* < 0.05).

In the production and processing of edible oils and fats, color could intuitively reflect the quality level. For PF1-6 and SP1-2, this indicator also had important indicative significance. The color of food was usually analyzed using the CIELab* color space because this parameter could evenly cover the entire visible spectrum to the human eye and could provide an intuitive and important reference dimension for its quality evaluation [55]. The colors of PF1-6 and SP1-2 were measured using the chromaticity L* (lightness), chromaticity a* (green-red), and chromaticity b* (blue-yellow) systems, and the Chroma (C*) was calculated according to the formula $C^{*}=\sqrt{{\left(a^{*}\right)}^{2}+{\left(b^{*}\right)}^{2}}$ (Figure 5B–E). The L* of oils, measured in the CIELab* color space, reflects their visual brightness, with higher values corresponding to a lighter and whiter appearance and lower values indicating a darker coloration. This parameter is primarily influenced by the composition and concentration of natural pigments, residual impurities, as well as products derived from thermal or oxidative reactions [56]. Among the analyzed samples, SP2 exhibited a notably higher L* value (39.99), which clearly distinguished it from the other samples. In contrast, the L* values of SP1, PF1, PF3, PF4, PF5, and PF6 clustered closely within the range of 34.00–34.95, showing no significant differences within this group. PF2 recorded the lowest L* value (29.35). The a* values of SP1 (−1.36), SP2 (−2.41), PF4 (−0.41), and PF5 (−0.32) were less than 0, indicating that these oils and fats were greenish; the b* values of all human milk fat substitutes were greater than 0, meaning that they were all yellowish. C* is a colorimetric parameter for the quantitative characterization of the vividness or saturation of oils and fats. As shown in Figure 5E, the C* values of SP1 and SP2 were 12.38 and 17.65, respectively, which were significantly lower than those of the PF series samples, whose values spanned a range of 19.50–27.66. Such chromatic differences could be associated with variations in raw material sources, disparities in processing technologies, or distinctions in oxidation and storage histories—all of which are factors known to modulate the chromophore composition of the final products. Notably, the instrumental chromatic data corroborate the visual observation that SP1 and SP2 exhibit lower color vividness compared with the PF samples (Figure 5A).

Lipid oxidation was one of the important factors leading to the decline in the sensory characteristics and nutritional value of oils and fats. In severe cases, compounds with potential toxicity would also be generated [57]. In this study, multiple indicators such as acid value, iodine value, saponification value, peroxide value, carbonyl value, *p*-anisidine value, and oxidation induction period of PF1-6 and SP1-2 were systematically evaluated to comprehensively assess their quality (Figure 5F–M). Acid value serves as a preliminary indicator of hydrolytic degradation, reflecting the level of free fatty acids. As shown in Figure 5F, the acid value of PF3 was measured at 3.30 mg KOH/g, a value significantly higher than those of all other samples, suggesting that PF3 may have undergone more extensive hydrolysis and thus exhibited inferior quality and freshness within the sample set. Iodine value characterizes the inherent degree of unsaturation. PF1 exhibited the highest iodine value (84.74 g I_2_/100 g, Figure 5G), indicating a greater proportion of unsaturated bonds and, consequently, a potentially higher susceptibility to oxidation [58]. In terms of saponification value, F3 and PF4 exhibited values of 197.55 mg KOH/g and 195.14 mg KOH/g, respectively (Figure 5H). Compared with the other PF fractions and SP, the higher saponification value of PF3 and PF4 suggests that the fatty acids in these fractions likely possess a lower average molecular weight and shorter average chain length. peroxide value can reflect the initial state of oil oxidation. At this stage, hydroperoxides gradually accumulate in the oil, leading to a continuous increase in the peroxide value [59]. revealed a notably high peroxide value of 44.54 mmol/kg for SP1 (Figure 5I). This value not only exceeded those of all other samples but also surpassed common acceptability thresholds for edible oils (9.85 mmol/kg, GB2716-2018), suggesting that SP1 may have experienced the most pronounced primary oxidation. Carbonyl value and *p*-anisidine value, markers of secondary oxidation, are also highest in SP1 (carbonyl value: 10.07 meq/kg, Figure 5J; *p*-anisidine value: 0.17), consistent with its elevated peroxide value. This pattern indicates a substantial accumulation of secondary carbonyl compounds, including specific Aldehydes such as 2-alkenals, in SP1 relative to other samples. The overall oxidation status was further evaluated using the total oxidation value (total oxidation value = 2 × peroxide value + *p*-anisidine value). SP1 exhibited the highest total oxidation value of 89.24 by a considerable margin. Together, the convergent results from peroxide value, carbonyl value, *p*-anisidine value, and total oxidation value consistently identify SP1 as the sample in the most advanced state of oxidative deterioration among those tested [60,61]. Under the same accelerated oxidation conditions, there were differences in the oxidation induction period performance of different PF and SP samples. As clearly shown in Figure 5M, the oxidation induction period of PF6 was as long as 37.84 h, significantly higher than that of the other human milk fat substitutes, indicating that PF6 might have relatively strong oxidation stability. It was worth noting that the oxidation induction period of all PF series samples was higher than that of the SP series, and the oxidation induction period of SP1 was at the lowest level, only 1.26 h. Surprisingly, the iodine value of PF1 and PF2 was significantly higher than that of the SP series; the actual detection results showed that their oxidation stability was significantly better than that of the SP series. This phenomenon might be due to the addition of butylated hydroxytoluene during the fish oil extraction process, and the addition amount conformed to the regulatory limit of ≤0.2 g/kg (GB 2760−2024) [62].

## 4. Conclusions

This study was a multidimensional study of PF1-6 and SP1-2 centered on the nutritional needs of infants and young children. Lipid characterization of the base oils revealed significant differences between animal and vegetable oils in terms of type and content of fatty acids and distribution of sn-2 fatty acids among the 20 base oils. Animal oils had higher levels of sn-2 LCSFA, while vegetable oils had higher levels of sn-2 n-esterified MUFA or PUFA. PF was output by MATLAB simulation. The total score of the PF series was higher than that of the SP series, with PF1 having the highest score. Multivariate statistical analysis showed that the HCA, PCA, and PLS-DA models all indicated that PF was closer to HMF than SP, which confirmed the feasibility of preparing human milk fat substitutes by simulation. For the quality evaluation of the complex lipids, the characterization of volatile organic compounds by GC-IMS revealed that there were differences in the types and contents of volatile organic compounds in PF1-6 and SP1-2, which could provide a basis for flavor modulation. The detection of the basic physicochemical indexes and oxidative stability showed that the acid value of PF3 was higher, the iodine value of PF1 was higher, the degree of oxidation of SP1 was the most serious, the oxidative stability of PF6 was stronger, and the overall oxidative stability of the PF series was better than that of the SP series. In conclusion, this study provides various references for optimizing human milk fat substitutes and meeting the nutritional requirements of infants and young children.

## Figures and Tables

**Table 1:** Scoring situations of six formulated lipids (PF) and two commercial products (SP).

Score	G_FA_	G_sn-2 FA_	G_TAG_	G
PF1	83.35	69.10	100.00	84.15
PF2	84.34	63.33	100.00	82.56
PF3	83.96	59.72	91.53	78.41
PF4	83.33	68.16	100.00	83.83
PF5	84.00	65.64	100.00	83.21
PF6	91.67	56.48	100.00	82.72
SP1	75.00	38.62	100.00	71.21
SP2	71.28	37.26	100.00	69.52

G_FA_ represents the scores for FA, G_sn-2 FA_ represents the scores for sn-2 FA, G_TAG_ represents the scores for TAG, and G represents the total score.

## Data Availability

The original contributions presented in the study are included in the article/Supplementary Material; further inquiries can be directed to the corresponding author.

## Supplementary Materials

The following supporting information can be downloaded at: https://www.mdpi.com/article/10.3390/foods15010081/s1.

## Abbreviations

The following abbreviations are used in this manuscript:

HMF	Human Milk Fat
PF	Formulated Lipid
SP	Commercial Products
TAG	Triglycerides
FA	Fatty Acids
sn-2FA	sn-2 Fatty Acids
OPO	1,3-dioleoyl-2-palmitoyl
OPL	1-oleoyl-2-palmitoyl-3-linoleoyl
VOCs	Volatile Organic Compounds
PUFA	Polyunsaturated Fatty Acids
DHA	Docosahexaenoic Acid
ARA	Arachidonic Acid
SFA	Saturated Fatty Acids
MUFA	Monounsaturated Fatty Acids
ALA	α-Linolenic Acid
EPA	Eicosapentaenoic Acid
GC-FID	Gas Chromatography-Flame Ionization Detector
