# Supplementary material for: Preparation of Human Milk Substitute Fat by Physical Blending and Its Quality Evaluation
Source: Foods. 2025 Dec 26;15(1):81. doi: 10.3390/foods15010081 (PMC12785833; doi:10.3390/foods15010081)
Supplement: Supplementary file 1 [file foods-15-00081-s001.zip › foods-4023673-supplementary.pdf]

Table S1 Total fatty acid (FA) compositions in 20 base oils (% of total fatty acids).

FA	Corn oil	Low erucic rapeseed oil	Flaxseed oil	Sunflower seed oil	Soybean oil	Coconut oil	Palm oil	Walnut oil	Rice bran oil	Peanut oil
C6:0	ND.	ND.	ND.	ND.	ND.	ND.	ND.	ND.	ND.	ND.
C8:0	ND.	ND.	ND.	ND.	ND.	0.77±0.60^{a}	ND.	ND.	ND.	ND.
C9:0	ND.	ND.	ND.	ND.	ND.	ND.	ND.	ND.	ND.	ND.
C10:0	ND.	ND.	ND.	ND.	ND.	4.11±0.9^{a}	0.03±0.01^{c}	ND.	ND.	ND.
C11:0	ND.	ND.	ND.	ND.	ND.	0.02±0.00^{b}	ND.	ND.	ND.	ND.
C12:0	ND.	0.02±0.00^{h}	ND.	ND.	ND.	50.63±0.25^{a}	0.42±0.01^{d}	ND.	0.02±0.00^{h}	ND.
C14:0	0.04±0.00^{i}	0.06±0.00^{i}	0.05±0.00^{i}	0.08±0.00^{hi}	0.07±0.03^{hi}	21.93±0.56^{a}	1.13±0.01^{g}	0.04±0.00^{i}	0.30±0.00^{h}	0.04±0.00^{i}
9cC14:1	ND.	ND.	ND.	ND.	ND.	ND.	ND.	ND.	ND.	ND.
C15:0	ND.	0.03±0.00^{i}	0.02±0.00^{ij}	0.02±0.00^{j}	ND.	ND.	0.04±0.00^{g}	0.02±0.00^{j}	0.04±0.00^{h}	ND.
C16:0	13.64±0.05^{k}	4.36±0.01^{s}	6.53±0.02^{q}	7.12±0.03^{p}	12.01±0.05^{m}	11.15±0.44^{n}	36.62±0.06^{b}	7.03±0.02^{p}	18.39±0.08^{j}	12.81±0.03^{l}
9tC16:1	ND.	ND.	ND.	ND.	ND.	ND.	ND.	ND.	ND.	ND.
9cC16:1	0.09±0.00^{kl}	0.20±0.00^{h}	0.08±0.01^{l}	0.10±0.00^{kl}	0.10±0.00^{kl}	ND.	0.22±0.01^{h}	0.11±0.00^{jk}	0.15±0.00^{i}	0.05±0.00^{m}
C17:0	0.07±0.00^{i}	0.11±0.00^{g}	0.06±0.00^{j}	0.03±0.00^{m}	0.10±0.00^{h}	ND.	0.08±0.00^{i}	0.05±0.00^{l}	0.05±0.00^{l}	0.08±0.00^{i}
10cC17:1	ND.	0.18±0.00^{bc}	ND.	ND.	0.12±0.11^{d}	ND.	ND.	ND.	ND.	ND.
C18:0	2.70±0.03^{q}	3.18±0.05^{p}	5.45±0.02^{i}	4.52±0.02^{l}	5.20±0.07^{j}	3.18±0.13^{p}	4.43±0.03^{m}	4.14±0.05^{n}	2.51±0.02^{r}	5.10±0.04^{k}
9tC18:1	0.40±0.00^{h}	0.30±0.05^{j}	0.23±0.00^{k}	0.37±0.01^{ij}	0.39±0.05^{hi}	ND.	0.33±0.01^{j}	0.36±0.01^{i}	0.68±0.02^{f}	0.67±0.02^{f}
11tC18:1	0.45±0.01^{g}	0.72±0.02^{b}	0.27±0.01^{j}	0.43±0.02^{h}	0.30±0.04^{i}	ND.	0.31±0.03^{i}	0.44±0.00^{gh}	ND.	ND.
6cC18:1	ND.	ND.	ND.	ND.	ND.	ND.	ND.	ND.	ND.	ND.
9cC18:1	25.49±0.03^{i}	69.37±0.04^{b}	17.99±0.01^{o}	25.28±0.02^{j}	23.64±0.04^{l}	6.67±0.48^{q}	41.68±0.05^{c}	21.58±0.05^{m}	36.15±0.01^{e}	41.54±0.03^{c}
11cC18:1	1.10±0.01^{m}	4.76±0.00^{b}	1.05±0.01^{m}	1.29±0.01^{l}	1.84±0.03^{j}	ND.	2.29±0.07^{h}	1.46±0.01^{k}	2.15±0.19^{i}	ND.
C19:0	ND.	ND.	ND.	ND.	ND.	ND.	ND.	ND.	ND.	ND.
9t12tC18:2n6	ND.	ND.	ND.	ND.	ND.	ND.	ND.	ND.	ND.	ND.
9c12cC18:2n6	54.72±0.01^{c}	13.38±0.03^{m}	15.69±0.01^{j}	60.17±0.08^{a}	50.19±0.12^{d}	1.54±0.15^{s}	11.85±0.01^{o}	56.53±0.24^{b}	37.35±0.13^{e}	36.89±0.02^{f}
C20:0	0.24±0.00^{f}	0.36±0.00^{c}	0.11±0.00^{j}	0.15±0.00^{i}	0.25±0.00^{f}	ND.	0.21±0.00^{g}	0.08±0.02^{k}	0.33±0.00^{d}	1.08±0.02^{a}
6c9c12cC18:3n6	ND.	ND.	ND.	ND.	ND.	ND.	ND.	0.25±0.28^{c}	ND.	ND.
5cC20:1	ND.	0.17±0.02^{a}	ND.	ND.	ND.	ND.	ND.	0.10±0.00^{c}	ND.	ND.
9c12c15cC18:3n3	1.01±0.01^{l}	1.10±0.01^{k}	52.47±0.05^{b}	0.23±0.00^{q}	5.69±0.03^{d}	ND.	0.37±0.03^{p}	7.13±0.04^{c}	1.78±0.00^{j}	0.66±0.01^{n}
8cC20:1	ND.	ND.	ND.	ND.	ND.	ND.	ND.	ND.	ND.	ND.
11cC20:1	ND.	1.53±0.02^{a}	ND.	ND.	ND.	ND.	ND.	0.63±0.00^{b}	0.06±0.00^{f}	ND.
C20:2n6	ND.	ND.	ND.	ND.	ND.	ND.	ND.	ND.	ND.	ND.
C22:0	ND.	0.08±0.00^{g}	ND.	0.21±0.00^{c}	ND.	ND.	ND.	ND.	ND.	ND.
C20:3n6	0.04±0.01^{i}	ND.	ND.	ND.	0.13±0.01^{e}	ND.	ND.	0.05±0.00^{hi}	0.06±0.00^{h}	0.91±0.03^{a}
C22:1n9	ND.	ND.	ND.	ND.	ND.	ND.	ND.	ND.	ND.	ND.
C20:3n3	ND.	ND.	ND.	ND.	ND.	ND.	ND.	ND.	ND.	ND.
C20:4n6	ND.	0.09±0.00^{h}	ND.	ND.	ND.	ND.	ND.	ND.	ND.	ND.
C22:2n6	ND.	ND.	ND.	ND.	ND.	ND.	ND.	ND.	ND.	ND.
C24:0	ND.	ND.	ND.	ND.	ND.	ND.	ND.	ND.	ND.	0.16±0.01^{a}
C22:4n6	ND.	ND.	ND.	ND.	ND.	ND.	ND.	ND.	ND.	ND.
C20:5n3	ND.	ND.	ND.	ND.	ND.	ND.	ND.	ND.	ND.	ND.
C24:1n9	ND.	ND.	ND.	ND.	ND.	ND.	ND.	ND.	ND.	ND.
C22:5n6	ND.	ND.	ND.	ND.	ND.	ND.	ND.	ND.	ND.	ND.
C22:5n3	ND.	ND.	ND.	ND.	ND.	ND.	ND.	ND.	ND.	ND.
C22:6n3	ND.	ND.	ND.	ND.	ND.	ND.	ND.	ND.	ND.	ND.
Σ SFA	16.69±0.03^{n}	8.20±0.05^{r}	12.22±0.05^{p}	12.14±0.04^{o}	17.61±0.11^{m}	91.79±0.63^{a}	42.95±0.04^{d}	11.37±0.05^{p}	21.62±0.07^{k}	19.28±0.04^{l}
Σ MUFA	27.54±0.03^{m}	77.23±0.03^{b}	19.61±0.01^{p}	27.46±0.04^{m}	26.38±0.09^{n}	6.67±0.48^{r}	44.82±0.02^{e}	24.67±0.06^{o}	39.19±0.20^{h}	42.26±0.01^{f}
Σ PUFA	55.77±0.01^{f}	14.57±0.03^{o}	68.16±0.05^{b}	60.40±0.08^{d}	56.01±0.15^{e}	1.54±0.15^{t}	12.22±0.02^{p}	63.96±0.01^{c}	39.19±0.14^{g}	38.46±0.03^{h}
Σ n-3	1.01±0.01^{l}	1.10±0.01^{l}	52.47±0.05^{b}	0.23±0.00^{q}	5.69±0.03^{d}	ND.	0.37±0.03^{p}	7.13±0.04^{c}	1.78±0.00^{k}	0.66±0.01^{o}
Σ n-6	54.76±0.02^{c}	13.47±0.03^{o}	15.69±0.01^{l}	60.17±0.08^{a}	50.32±0.12^{d}	1.54±0.15^{t}	11.85±0.01^{p}	56.84±0.04^{b}	37.40±0.13^{f}	37.80±0.02^{e}
Σ TFA	0.86±0.01^{j}	1.01±0.02^{h}	0.49±0.01^{o}	0.79±0.01^{k}	0.69±0.01^{l}	ND.	0.64±0.02^{m}	0.79±0.01^{k}	0.68±0.02^{l}	0.67±0.02^{l}

Table S1 Continued.

FA	Perilla seed oil	Camellia oil	Butterfat	Lard	Tilapia solid fat	Tilapia liquid oil	Basa catfish solid fat	Basa catfish liquid oil	Golden pompano solid fat	Golden pompano liquid oil
C6:0	ND.	ND.	ND.	0.23±0.02^{a}	ND.	ND.	ND.	ND.	ND.	ND.
C8:0	ND.	ND.	0.11±0.14^{b}	ND.	ND.	ND.	ND.	ND.	ND.	ND.
C9:0	ND.	ND.	0.03±0.02^{a}	ND.	ND.	ND.	ND.	ND.	ND.	ND.
C10:0	0.04±0.01^{c}	ND.	2.15±0.53^{b}	0.07±0.01^{c}	ND.	ND.	ND.	ND.	ND.	ND.
C11:0	ND.	ND.	0.11±0.01^{a}	ND.	ND.	ND.	ND.	ND.	ND.	ND.
C12:0	0.32±0.02^{e}	0.02±0.00^{h}	3.83±0.08^{b}	0.10±0.00^{fgh}	0.15±0.00^{fg}	0.20±0.00^{f}	0.04±0.00^{h}	0.60±0.04^{c}	0.04±0.00^{h}	0.04±0.00^{gh}
C14:0	0.17±0.00^{hi}	0.12±0.00^{hi}	12.15±0.05^{b}	1.61±0.01^{f}	2.96±0.02^{d}	2.84±0.01^{d}	1.89±0.02^{e}	4.43±0.01^{c}	1.89±0.03^{e}	1.85±0.02^{e}
9cC14:1	ND.	ND.	1.02±0.01^{a}	ND.	0.08±0.00^{c}	0.13±0.00^{b}	0.03±0.00^{f}	0.06±0.00^{d}	0.03±0.00^{f}	0.03±0.00^{e}
C15:0	0.02±0.00^{j}	ND.	1.37±0.01^{a}	0.04±0.00^{h}	0.34±0.01^{b}	0.27±0.00^{c}	0.25±0.00^{d}	0.16±0.00^{f}	0.25±0.00^{d}	0.23±0.00^{e}
C16:0	5.75±0.01^{r}	9.03±0.02^{o}	36.84±0.27^{a}	28.94±0.09^{c}	27.49±0.06^{d}	19.55±0.01^{i}	26.07±0.02^{e}	20.03±0.06^{h}	25.70±0.21^{f}	21.27±0.08^{g}
9tC16:1	ND.	ND.	0.36±0.00^{b}	ND.	0.37±0.00^{a}	0.33±0.02^{c}	0.13±0.00^{d}	0.11±0.01^{e}	0.13±0.00^{d}	0.13±0.00^{d}
9cC16:1	0.06±0.00^{m}	0.13±0.00^{j}	2.27±0.03^{f}	2.15±0.01^{g}	4.00±0.02^{b}	5.39±0.01^{a}	2.81±0.01^{d}	2.53±0.01^{e}	2.79±0.03^{d}	3.32±0.01^{c}
C17:0	0.06±0.00^{jk}	0.05±0.00^{kl}	0.67±0.01^{a}	0.21±0.00^{e}	0.43±0.00^{b}	0.25±0.00^{d}	0.28±0.00^{c}	0.13±0.02^{f}	0.27±0.00^{c}	0.21±0.00^{e}
10cC17:1	ND.	0.06±0.00^{e}	0.30±0.00^{a}	0.14±0.00^{cd}	0.20±00^{b}	0.26±0.00^{a}	0.14±0.00^{cd}	0.15±0.00^{cd}	0.14±0.00^{cd}	0.17±0.00^{bc}
C18:0	4.44±0.01^{lm}	3.63±0.00^{o}	9.98±0.10^{b}	15.72±0.04^{a}	8.90±0.05^{c}	5.85±0.04^{h}	7.39±0.03^{d}	6.59±0.01^{f}	7.22±0.07^{e}	5.95±0.04^{g}
9tC18:1	0.40±0.00^{hi}	0.93±0.01^{c}	1.25±0.02^{a}	0.57±0.00^{g}	0.96±0.02^{c}	1.13±0.01^{b}	0.78±0.01^{de}	0.95±0.02^{c}	0.75±0.01^{e}	0.80±0.01^{d}
11tC18:1	ND.	ND.	0.83±0.01^{a}	ND.	0.50±0.01^{f}	0.57±0.01^{d}	0.56±0.00^{d}	0.52±0.03^{e}	0.56±0.01^{d}	0.61±0.01^{c}
6cC18:1	ND.	ND.	0.44±0.01^{a}	ND.	ND.	ND.	ND.	ND.	ND.	ND.
9cC18:1	14.68±0.01^{p}	77.68±0.03^{a}	21.15±0.20^{n}	36.05±0.04^{e}	29.33±0.02^{g}	34.03±0.05^{f}	24.71±0.00^{k}	41.24±0.02^{d}	24.61±0.05^{k}	26.2±0.04^{h}
11cC18:1	1.03±0.01^{m}	ND.	1.34±0.02^{l}	3.56±0.01^{e}	4.37±0.01^{c}	4.91±0.02^{a}	3.27±0.01^{g}	4.23±0.02^{d}	3.33±0.00^{g}	3.45±0.01^{f}
C19:0	ND.	ND.	ND.	ND.	0.06±0.00^{a}	0.04±0.01^{b}	ND.	ND.	0.04±0.00^{b}	ND.
9t12tC18:2n6	0.10±0.00^{b}	ND.	0.19±0.01^{a}	ND.	0.02±0.00^{d}	0.06±0.00^{c}	ND.	ND.	ND.	ND.
9c12cC18:2n6	14.42±0.01^{k}	7.70±0.01^{p}	2.92±0.03^{r}	9.36±0.01^{p}	13.05±0.02^{n}	16.29±0.04^{i}	24.11±0.02^{h}	13.87±0.01^{l}	24.04±0.09^{h}	27.21±0.05^{g}
C20:0	ND.	0.11±0.00^{j}	0.08±0.00^{k}	0.15±0.00^{i}	0.32±0.01^{d}	0.24±0.01^{f}	0.43±0.00^{b}	0.18±0.00^{h}	0.42±0.01^{b}	0.31±0.00^{e}
6c9c12cC18:3n6	ND.	ND.	ND.	ND.	0.95±0.00^{b}	1.29±0.00^{a}	ND.	0.23±0.00^{c}	ND.	ND.
5cC20:1	0.13±0.00^{b}	ND.	ND.	ND.	ND.	ND.	ND.	ND.	ND.	ND.
9c12c15cC18:3n3	58.00±0.02^{a}	0.43±0.00^{o}	0.37±0.00^{p}	0.74±0.01^{m}	2.33±0.02^{h}	2.56±0.02^{g}	3.15±0.01^{f}	2.17±0.01^{i}	3.16±0.03^{f}	3.24±0.02^{e}
8cC20:1	ND.	ND.	ND.	ND.	ND.	ND.	ND.	ND.	0.08±0.00^{b}	0.08±0.00^{a}
11cC20:1	0.35±0.01^{c}	ND.	ND.	ND.	ND.	ND.	ND.	ND.	0.15±0.00^{d}	0.13±0.00^{e}
C20:2n6	ND.	ND.	ND.	0.28±0.00^{e}	0.56±0.01^{c}	0.57±0.00^{c}	1.41±0.01^{a}	0.44±0.00^{d}	1.41±0.03^{a}	1.37±0.03^{b}
C22:0	ND.	ND.	0.10±0.00^{ef}	ND.	0.10±0.00^{e}	0.09±0.00f	0.24±0.00^{b}	0.05±0.00^{h}	0.25±0.02^{a}	0.17±0.01^{d}
C20:3n6	ND.	0.13±0.00^{e}	ND.	ND.	0.54±0.00^{d}	0.66±0.00^{b}	0.08±0.00^{g}	0.60±0.00^{c}	0.08±0.00^{g}	0.10±0.00^{f}
C22:1n9	ND.	ND.	ND.	ND.	ND.	ND.	0.24±0.00^{b}	ND.	0.26±0.02^{a}	0.18±0.01^{d}
C20:3n3	0.04±0.00^{g}	ND.	ND.	ND.	0.18±0.00^{d}	0.17±0.00^{e}	0.25±0.00^{b}	0.10±0.00^{f}	0.26±0.01^{a}	0.24±0.00^{c}
C20:4n6	ND.	ND.	0.14±0.00^{g}	0.07±0.00^{i}	0.60±0.00^{b}	0.81±0.00^{a}	0.16±0.01^{f}	0.25±0.00^{d}	0.17±0.00^{e}	0.26±0.00^{c}
C22:2n6	ND.	ND.	ND.	ND.	ND.	ND.	ND.	ND.	0.16±0.01^{a}	0.12±0.01^{b}
C24:0	ND.	ND.	ND.	ND.	ND.	ND.	ND.	ND.	0.04±0.00^{b}	0.02±0.00^{c}
C22:4n6	ND.	ND.	ND.	ND.	0.32±0.01^{b}	0.39±0.01^{a}	0.05±0.00^{e}	0.08±0.00^{c}	0.05±0.00^{e}	0.06±0.00^{d}
C20:5n3	ND.	ND.	ND.	ND.	0.07±0.00^{d}	0.09±0.00^{c}	0.25±0.00^{b}	0.06±0.00^{e}	0.25±0.00^{b}	0.33±0.01^{a}
C24:1n9	ND.	ND.	ND.	ND.	ND.	ND.	ND.	ND.	0.07±0.01^{a}	0.04±0.00^{b}
C22:5n6	ND.	ND.	ND.	ND.	0.32±0.01^{b}	0.39±0.01^{a}	0.08±0.00^{e}	0.09±0.01d	0.10±0.01^{d}	0.12±0.00^{c}
C22:5n3	ND.	ND.	ND.	ND.	0.17±0.00^{e}	0.21±0.00^{d}	0.27±0.00^{c}	0.06±0.00^{f}	0.29±0.02^{b}	0.31±0.01^{a}
C22:6n3	ND.	ND.	ND.	ND.	0.33±0.01^{e}	0.44±0.01^{d}	0.94±0.01^{c}	0.09±0.00^{f}	0.99±0.05^{b}	1.44±0.05^{a}
Σ SFA	10.8±0.01^{q}	12.96±0.02^{o}	67.42±0.32^{b}	47.07±0.05^{c}	40.75±0.07^{e}	29.33±0.04^{j}	36.58±0.05^{f}	32.17±0.02^{h}	36.14±0.17^{g}	30.05±0.12^{i}
Σ MUFA	16.64±0.00^{q}	78.79±0.03^{a}	28.96±0.28^{l}	42.47±0.03^{f}	39.82±0.01^{g}	46.76±0.01^{d}	32.67±0.02^{k}	49.79±0.03^{c}	32.91±0.03^{j}	35.15±0.03^{i}
Σ PUFA	72.56±0.01^{a}	8.25±0.01^{r}	3.62±0.04^{s}	10.45±0.02^{q}	19.43±0.06^{m}	23.91±0.04^{l}	30.74±0.04^{k}	18.04±0.00^{n}	30.95±0.15^{j}	34.80±0.13^{i}
Σ n-3	58.04±0.02^{a}	0.43±0.00^{p}	0.37±0.00^{p}	0.74±0.01^{m}	3.08±0.02^{i}	3.46±0.01^{h}	4.86±0.02^{g}	2.48±0.01^{j}	4.94±0.11^{f}	5.56±0.08^{e}
Σ n-6	14.52±0.01^{n}	7.82±0.01^{r}	3.25±0.04^{s}	9.71±0.02^{q}	16.35±0.03^{k}	20.45±0.03^{j}	25.89±0.02^{i}	15.56±0.01^{m}	26.01±0.08^{h}	29.24±0.06^{g}
Σ TFA	0.50±0.00^{o}	0.93±0.01^{i}	2.63±0.03^{a}	0.57±0.00^{n}	1.85±0.02^{c}	2.09±0.03^{b}	1.47±0.01^{f}	1.59±0.00^{d}	1.44±0.02^{g}	1.54±0.02^{e}

Results are means ±standard deviation (n=3). Values in the same row with different letters are significantly different (p< 0.05). "ND." stands for "not detected".

Table S2 Composition of sn-2 fatty acids (sn-2 FA) in 20 base oils (% of total fatty acids).

sn-2 FA	Corn oil	Low erucic rapeseed oil	Flaxseed oil	Sunflower oil	Soybean oil	Coconut oil	Palm oil	Walnut oil	Rice bran oil	Peanut oil
C6:0	ND.	ND.	ND.	ND.	ND.	ND.	ND.	ND.	ND.	ND.
C8:0	ND.	ND.	ND.	ND.	ND.	ND.	ND.	ND.	ND.	ND.
C9:0	ND.	ND.	ND.	ND.	ND.	ND.	ND.	ND.	ND.	ND.
C10:0	ND.	ND.	ND.	ND.	ND.	1.07±0.04^{a}	0.06±0.02^{d}	ND.	ND.	ND.
C11:0	ND.	ND.	ND.	ND.	ND.	ND.	ND.	ND.	ND.	ND.
C12:0	ND.	0.24±0.01^{def}	ND.	ND.	ND.	65.22±0.89^{a}	0.43±0.04^{d}	ND.	0.17±0.01^{def}	ND.
C14:0	0.09±0.01^{l}	0.25±0.01^{jk}	0.17±0.03^{kl}	0.32±0.02^{j}	0.16±0.03^{kl}	10.71±0.27^{b}	0.59±0.05^{i}	0.60±0.00^{i}	0.18±0.02^{kl}	0.16±0.02^{kl}
9cC14:1	ND.	ND.	ND.	ND.	ND.	ND.	ND.	ND.	ND.	ND.
C15:0	ND.	0.13±0.05^{f}	0.06±0.01^{ghi}	0.08±0.00^{fgh}	ND.	ND.	0.05±0.00^{hij}	0.11±0.00^{fg}	0.06±0.01^{hij}	ND.
C16:0	3.96±0.06^{o}	5.54±0.03^{n}	5.29±0.02^{n}	12.45±0.04^{j}	3.31±0.02^{p}	9.66±0.23^{k}	15.47±0.04^{i}	7.33±0.01^{l}	6.29±0.01^{m}	3.73±0.03^{op}
9tC16:1	ND.	ND.	ND.	ND.	ND.	ND.	ND.	ND.	ND.	ND.
9cC16:1	0.11±0.00^{ij}	0.13±0.00^{i}	0.11±0.00^{ij}	0.06±0.00^{ij}	0.11±0.00^{ij}	ND.	0.15±0.00^{i}	0.14±0.00^{i}	0.13±0.00^{i}	0.05±0.00^{ij}
C17:0	ND.	0.03±0.00^{h}	0.14±0.00^{f}	0.06±0.00^{g}	ND.	ND.	0.04±0.00^{gh}	0.05±0.00^{gh}	ND.	0.04±0.03^{gh}
10cC17:1	ND.	0.17±0.00^{b}	ND.	ND.	0.07±0.00^{e}	ND.	ND.	ND.	ND.	ND.
C18:0	2.60±0.15^{m}	4.19±0.02^{j}	4.22±0.06^{j}	9.42±0.03^{e}	2.28±0.04^{n}	6.29±0.16^{h}	4.86±0.02^{i}	3.69±0.02^{k}	2.62±0.00^{m}	2.29±0.02^{n}
9tC18:1	0.54±0.06^{c}	0.34±0.01^{fg}	0.37±0.04^{ef}	0.32±0.03^{fgh}	0.46±0.02^{d}	ND.	0.26±0.03^{h}	0.18±0.02^{i}	0.55±0.01^{c}	0.42±0.02^{de}
11tC18:1	0.15±0.02^{f}	0.40±0.01^{b}	0.21±0.05^{e}	0.12±0.01^{g}	0.14±0.02^{f}	ND.	0.38±0.02^{c}	0.26±0.03^{d}	ND.	ND.
6cC18:1	ND.	ND.	ND.	ND.	ND.	ND.	ND.	ND.	ND.	ND.
9cC18:1	25.02±0.02^{g}	65.83±0.01^{b}	23.29±0.02^{hi}	25.26±0.02^{g}	26.66±0.04^{f}	5.34±0.15^{n}	58.71±0.05^{c}	24.33±0.11^{gh}	38.36±0.00^{d}	36.27±0.04^{e}
11cC18:1	ND.	ND.	ND.	ND.	ND.	ND.	ND.	0.86±0.10^{g}	ND.	ND.
9t12tC18:2n6	ND.	ND.	ND.	ND.	ND.	ND.	ND.	ND.	ND.	ND.
9c12cC18:2n6	66.32±0.25^{a}	18.57±0.01^{g}	20.19±0.09^{f}	51.39±0.06^{d}	61.91±0.09^{b}	1.71±0.06^{o}	18.68±0.04^{g}	56.20±0.10^{c}	49.89±0.02^{e}	56.58±0.07^{c}
C20:0	ND.	0.08±0.00^{cd}	ND.	0.13±0.00^{a}	ND.	ND.	0.07±0.00^{de}	0.05±0.00^{ef}	0.08±0.00^{c}	0.07±0.00^{cd}
6c9c12cC18:3n6	ND.	ND.	ND.	ND.	ND.	ND.	ND.	0.19±0.00^{c}	ND.	ND.
5cC20:1	ND.	0.22±0.00^{a}	ND.	ND.	ND.	ND.	ND.	0.07±0.00^{c}	ND.	ND.
9c12c15cC18:3n3	0.87±0.01^{g}	ND.	45.94±0.07^{b}	0.16±0.00^{ij}	4.62±0.01^{d}	ND.	0.25±0.00^{i}	5.35±0.01^{c}	1.49±0.00^{e}	0.16±0.00^{ij}
11cC20:1	ND.	2.13±0.00^{a}	ND.	ND.	ND.	ND.	ND.	0.45±0.00^{b}	0.07±0.00^{d}	ND.
C20:2n6	ND.	ND.	ND.	ND.	ND.	ND.	ND.	ND.	ND.	ND.
C22:0	ND.	0.08±0.05^{c}	ND.	0.23±0.00^{a}	ND.	ND.	ND.	ND.	ND.	ND.
C20:3n6	0.34±0.03^{a}	ND.	ND.	ND.	0.29±0.00^{b}	ND.	ND.	0.13±0.03^{e}	0.13±0.00^{e}	0.14±0.00^{e}
C22:1n9	ND.	ND.	ND.	ND.	ND.	ND.	ND.	ND.	ND.	ND.
C20:3n3	ND.	ND.	ND.	ND.	ND.	ND.	ND.	ND.	ND.	ND.
C20:4n6	ND.	1.68±0.03^{c}	ND.	ND.	ND.	ND.	ND.	ND.	ND.	ND.
C22:2n6	ND.	ND.	ND.	ND.	ND.	ND.	ND.	ND.	ND.	ND.
C24:0	ND.	ND.	ND.	ND.	ND.	ND.	ND.	ND.	ND.	0.09±0.02^{a}
C22:4n6	ND.	ND.	ND.	ND.	ND.	ND.	ND.	ND.	ND.	ND.
C20:5n3	ND.	ND.	ND.	ND.	ND.	ND.	ND.	ND.	ND.	ND.
C22:5n6	ND.	ND.	ND.	ND.	ND.	ND.	ND.	ND.	ND.	ND.
C22:5n3	ND.	ND.	ND.	ND.	ND.	ND.	ND.	ND.	ND.	ND.
C22:6n3	ND.	ND.	ND.	ND.	ND.	ND.	ND.	ND.	ND.	ND.
Σ SFA	6.65±0.21^{p}	10.53±0.04^{m}	9.90±0.09^{n}	22.69±0.07^{j}	5.75±0.05^{q}	92.95±0.2^{a}	21.56±0.06^{k}	11.82±0.02^{l}	9.40±0.02^{no}	6.39±0.10^{p}
Σ MUFA	25.82±0.06^{g}	69.22±0.02^{b}	23.98±0.04^{h}	25.76±0.02^{g}	27.44±0.05^{f}	5.34±0.15^{n}	59.51±0.05^{c}	26.30±0.11^{fg}	39.1±0.01^{d}	36.74±0.02^{e}
Σ PUFA	67.53±0.26^{b}	20.25±0.04^{i}	66.13±0.08^{d}	51.55±0.05^{g}	66.81±0.10^{c}	1.71±0.06^{s}	18.94±0.04^{j}	61.88±0.09^{e}	51.51±0.02^{g}	56.88±0.08^{f}
Σ n-3	0.87±0.01^{l}	ND.	45.94±0.07^{b}	0.16±0.00^{no}	4.62±0.01^{d}	ND.	0.25±0.00^{n}	5.35±0.01^{c}	1.49±0.00^{j}	0.16±0.00^{no}
Σ n-6	66.66±0.26^{a}	20.25±0.04^{f}	20.19±0.09^{f}	51.39±0.06^{d}	62.20±0.09^{b}	1.71±0.06^{o}	18.68±0.04^{g}	56.52±0.08^{c}	50.01±0.02^{e}	56.72±0.08^{c}
Σ TFA	0.69±0.04^{de}	0.74±0.01^{cd}	0.58±0.02^{g}	0.43±0.02^{i}	0.60±0.01^{fg}	ND.	0.64±0.05^{ef}	0.44±0.02^{i}	0.55±0.01^{gh}	0.42±0.02^{i}

Table S2 Continued.

sn-2 FA	Perilla seed oil	Camellia oil	Butterfat	Lard	Tilapia solid fat	Tilapia liquid oil	Basa catfish solid fat	Basa catfish liquid oil	Golden pompano solid fat	Golden pompano liquid oil
C6:0	ND.	ND.	ND.	0.15±0.00^{a}	ND.	ND.	ND.	ND.	ND.	ND.
C8:0	ND.	ND.	0.04±0.02^{a}	ND.	ND.	ND.	ND.	ND.	ND.	ND.
C9:0	ND.	ND.	0.02±0.01^{a}	ND.	ND.	ND.	ND.	ND.	ND.	ND.
C10:0	0.07±0.02^{d}	ND.	0.71±0.11^{b}	0.19±0.01^{c}	ND.	ND.	ND.	ND.	ND.	ND.
C11:0	ND.	ND.	0.07±0.00^{a}	ND.	ND.	ND.	ND.	ND.	ND.	ND.
C12:0	0.11±0.04^{ef}	0.11±0.05^{ef}	3.39±0.08^{b}	0.39±0.02^{de}	0.23±0.01^{def}	0.41±0.04^{d}	1.04±0.05^{c}	0.88±0.04^{c}	0.23±0.02^{def}	0.29±0.02^{def}
C14:0	0.08±0.02^{l}	0.19±0.09^{kl}	15.30±0.03^{a}	3.58±0.05^{e}	3.65±0.03^{e}	3.24±0.05^{f}	5.19±0.04^{c}	4.32±0.01^{d}	2.67±0.06^{g}	2.43±0.05^{h}
9cC14:1	ND.	ND.	0.72±0.00^{a}	ND.	0.07±0.02^{e}	0.14±0.02^{b}	0.08±0.03^{de}	0.12±0.04^{cd}	0.08±0.01^{de}	0.11±0.01^{cd}
C15:0	0.02±0.00^{ij}	ND.	1.40±0.00^{a}	0.13±0.00^{f}	0.47±0.03^{b}	0.45±0.09^{bc}	0.31±0.03^{d}	0.24±0.04^{e}	0.40±0.03^{c}	0.31±0.00^{d}
C16:0	2.09±0.05^{q}	5.38±1.86^{n}	43.81±0.13^{g}	66.87±0.02^{a}	55.42±0.34^{c}	49.00±0.31^{f}	57.59±0.10^{b}	42.99±0.07^{h}	49.84±0.28^{e}	50.86±0.15^{d}
9tC16:1	ND.	ND.	0.34±0.01^{a}	ND.	0.31±0.01^{b}	0.23±0.01^{c}	0.15±0.02^{d}	0.12±0.02^{e}	0.12±0.00^{e}	0.24±0.00^{c}
9cC16:1	0.07±0.00^{ij}	0.27±0.35^{h}	2.26±0.07^{cd}	2.35±0.00^{c}	2.60±0.03^{b}	2.92±0.03^{a}	1.41±0.03^{g}	1.89±0.05^{e}	2.22±0.02^{d}	1.70±0.02^{f}
C17:0	ND.	0.03±0.02^{h}	0.58±0.01^{a}	0.30±0.00^{d}	0.52±0.00^{b}	0.35±0.03^{c}	0.24±0.02^{e}	0.15±0.01^{f}	0.32±0.00^{d}	0.23±0.00^{e}
10cC17:1	ND.	0.06±0.02^{e}	0.26±0.01^{a}	0.13±0.00^{c}	0.14±0.00^{c}	0.16±0.00^{b}	0.10±0.02^{d}	0.11±0.01^{d}	0.10±0.01^{d}	ND.
C18:0	1.54±0.01^{o}	3.15±0.20^{l}	10.82±0.04^{c}	9.68±0.01^{d}	7.42±0.21^{g}	12.08±0.27^{b}	9.04±0.16^{f}	9.86±0.14^{d}	9.38±0.10^{e}	17.1±0.12^{a}
9tC18:1	0.26±0.01^{h}	0.74±0.08^{a}	0.30±0.02^{gh}	0.18±0.00^{i}	0.57±0.05^{c}	0.48±0.06^{d}	0.37±0.02^{ef}	0.63±0.03^{b}	0.44±0.04^{d}	ND.
11tC18:1	ND.	ND.	1.22±0.01^{a}	ND.	ND.	ND.	ND.	ND.	ND.	ND.
6cC18:1	ND.	ND.	0.46±0.00^{a}	ND.	ND.	ND.	ND.	ND.	ND.	ND.
9cC18:1	ND.	79.02±4.43^{a}	14.65±0.05^{j}	10.98±0.01^{l}	12.59±0.06^{k}	13.14±0.02^{k}	15.39±0.04^{j}	22.05±0.10^{i}	10.85±0.04^{l}	8.43±0.03^{m}
11cC18:1	19.22±0.01^{a}	ND.	0.75±0.00^{i}	0.81±0.00^{h}	2.37±0.03^{b}	2.10±0.02^{d}	1.74±0.04^{e}	2.21±0.05^{c}	1.16±0.03^{f}	0.78±0.01^{hi}
9t12tC18:2n6	0.08±0.00^{b}	ND.	0.12±0.00^{a}	ND.	ND.	ND.	ND.	ND.	ND.	ND.
9c12cC18:2n6	20.37±0.11^{f}	10.42±1.44^{j}	2.04±0.01^{o}	2.85±0.01^{n}	8.64±0.02^{l}	9.04±0.03^{l}	4.62±0.02^{m}	9.57±0.05^{k}	16.22±0.03^{h}	11.04±0.03^{i}
C20:0	ND.	0.07±0.03^{d}	0.07±0.00^{cd}	0.13±0.00^{a}	0.07±0.01^{cd}	ND.	0.05±0.00^{ef}	ND.	0.11±0.02^{b}	ND.
6c9c12cC18:3n6	ND.	ND.	ND.	ND.	0.53±0.01^{b}	0.67±0.01^{a}	0.05±0.01^{f}	0.18±0.01^{e}	ND.	ND.
5cC20:1	0.10±0.01^{b}	ND.	ND.	ND.	ND.	ND.	ND.	ND.	ND.	ND.
9c12c15cC18:3n3	55.61±0.21^{a}	0.51±0.49^{h}	0.16±0.00^{ij}	0.25±0.00^{i}	1.15±0.01^{f}	1.10±0.01^{f}	0.60±0.00^{h}	1.07±0.00^{f}	1.37±0.00^{e}	0.91±0.00^{g}
11cC20:1	0.31±0.00^{c}	ND.	ND.	ND.	ND.	ND.	ND.	ND.	ND.	ND.
C20:2n6	ND.	ND.	ND.	0.40±0.01^{b}	0.24±0.01^{d}	0.21±0.00^{e}	0.20±0.01^{f}	0.57±0.01^{a}	0.26±0.00^{c}	0.18±0.01^{g}
C22:0	ND.	ND.	0.04±0.00^{d}	ND.	0.07±0.02^{c}	0.08±0.01^{c}	0.03±0.00^{d}	0.07±0.00^{c}	0.10±0.01^{b}	0.11±0.00^{b}
C20:3n6	ND.	0.05±0.01^{g}	ND.	ND.	0.18±0.01^{d}	ND.	0.08±0.00^{f}	0.24±0.01^{c}	0.07±0.02^{f}	ND.
C22:1n9	ND.	ND.	ND.	ND.	ND.	ND.	ND.	ND.	0.11±0.00^{a}	ND.
C20:3n3	0.06±0.00^{e}	ND.	ND.	ND.	0.72±0.05^{c}	1.17±0.07^{b}	0.51±0.02^{d}	0.75±0.01^{c}	0.73±0.01^{c}	1.70±0.06^{a}
C20:4n6	ND.	ND.	0.46±0.00^{j}	0.62±0.01^{i}	0.68±0.00^{h}	1.86±0.01^{b}	1.09±0.00^{g}	1.52±0.02^{e}	1.23±0.01^{f}	2.60±0.03^{a}
C22:2n6	ND.	ND.	ND.	ND.	ND.	ND.	ND.	ND.	0.06±0.03^{a}	ND.
C24:0	ND.	ND.	ND.	ND.	ND.	ND.	ND.	ND.	ND.	ND.
C22:4n6	ND.	ND.	ND.	ND.	0.33±0.02^{a}	0.32±0.01^{b}	ND.	0.09±0.00^{c}	ND.	ND.
C20:5n3	ND.	ND.	ND.	ND.	0.05±0.02^{b}	ND.	ND.	ND.	0.17±0.01^{a}	0.17±0.03^{a}
C22:5n6	ND.	ND.	ND.	ND.	0.24±0.01^{a}	0.22±0.01^{b}	0.03±0.00^{e}	0.10±0.01^{c}	0.06±0.01^{d}	ND.
C22:5n3	ND.	ND.	ND.	ND.	0.25±0.01^{b}	0.23±0.01^{bc}	0.05±0.03^{e}	0.11±0.00^{d}	0.36±0.00^{a}	0.21±0.05^{c}
C22:6n3	ND.	ND.	ND.	ND.	0.47±0.02^{c}	0.42±0.02^{d}	0.04±0.01^{f}	0.15±0.00^{e}	1.31±0.02^{a}	0.63±0.01^{b}
Σ SFA	3.93±0.13^{r}	8.93±2.16^{o}	76.27±0.08^{c}	81.42±0.03^{b}	67.85±0.19^{f}	65.59±0.06^{g}	73.48±0.09^{d}	58.50±0.03^{i}	63.06±0.14^{h}	71.32±0.07^{e}
Σ MUFA	19.96±0.01^{ij}	80.09±4.08^{a}	20.96±0.07^{i}	14.46±0.02^{l}	18.66±0.14^{k}	19.17±0.07^{jk}	19.25±0.07^{jk}	27.14±0.07^{f}	15.08±0.09^{l}	11.26±0.04^{m}
Σ PUFA	76.11±0.13^{a}	10.98±1.92^{o}	2.77±0.01^{r}	4.12±0.02^{q}	13.49±0.11^{n}	15.24±0.10^{l}	7.27±0.03^{p}	14.36±0.09^{m}	21.86±0.05^{h}	17.42±0.06^{k}
Σ n-3	55.66±0.21^{a}	0.51±0.49^{m}	0.16±0.00^{no}	0.25±0.00^{n}	2.64±0.10^{h}	2.91±0.10^{g}	1.20±0.04^{k}	2.08±0.01^{i}	3.95±0.05^{e}	3.61±0.07^{f}
Σ n-6	20.45±0.11^{f}	10.47±1.44^{k}	2.62±0.01^{n}	3.87±0.02^{m}	10.85±0.04^{k}	12.32±0.05^{j}	6.07±0.02^{l}	12.27±0.08^{j}	17.9±0.03^{h}	13.81±0.05^{i}
Σ TFA	0.35±0.01^{j}	0.74±0.08^{cd}	1.98±0.03^{a}	0.18±0.00^{l}	0.88±0.06^{b}	0.71±0.06^{cd}	0.52±0.01^{h}	0.75±0.03^{c}	0.56±0.04^{gh}	0.24±0.00^{k}

Results are means ±standard deviation (n=3). Values in the same row with different letters are significantly different ($p < 0.05$). “ND.” stands for “not detected”.

Table S3 Base oil ratio.

Base oil/%	PF1	PF2	PF3	PF4	PF5	PF6
Corn oil	10.00	ND.	ND.	7.28	10.00	0.23
Low erucic rapeseed oil	5.00	ND.	2.00	ND.	ND.	10.00
Flaxseed oil	10.00	ND.	ND.	1.00	ND.	ND.
Sunflower seed oil	1.20	11.99	3.60	ND.	2.00	ND.
Soybean oil	ND.	5.00	ND.	ND.	2.00	ND.
Coconut oil	10.51	10.68	7.69	9.12	10.64	10.43
Palm oil	ND.	5.11	ND.	4.00	7.70	ND.
Walnut oil	ND.	5.00	ND.	ND.	ND.	5.00
Rice bran oil	ND.	ND.	1.00	ND.	ND.	ND.
Peanut oil	ND.	ND.	1.00	ND.	ND.	ND.
Perilla seed oil	4.79	6.47	1.97	3.10	2.52	1.66
Camellia oil	ND.	ND.	ND.	ND.	0.17	ND.
Butterfat	5.74	ND.	55.00	3.00	ND.	5.83
Lard	ND.	50.31	ND.	35.49	44.96	ND.
Tilapia solid fat	62.84	ND.	ND.	ND.	ND.	ND.
Tilapia liquid oil	ND.	5.45	ND.	ND.	ND.	ND.
Basa catfish solid fat	ND.	ND.	30.98	ND.	ND.	ND.
Basa catfish liquid oil	ND.	ND.	ND.	10.00	ND.	ND.
Golden pompano solid fat	ND.	ND.	ND.	ND.	ND.	20.00
Golden pompano liquid oil	ND.	ND.	ND.	ND.	20.00	46.85

“ND.” It indicates that the base oil has not been added.

Table S4 Volatile organic compounds of six formulated lipids (PF) and two commercial products (SP)

Compounds	CAS	Formula	Flavor Profile	Content (%)							
				PF1	PF2	PF3	PF4	PF5	PF6	SP1	SP2
**Alcohols**											
4-Methyl-1-pentanol(M)	C626891	C6H14O	Sweet, Fruit, Alcohol	3.03±0.08^{a}	0.14±0.02^{de}	0.09±0.01^{de}	0.06±0.01^{e}	0.44±0.02^{c}	0.51±0.01^{b}	0.06±0.01^{e}	0.12±0.03^{d}
4-Methyl-1-pentanol(D)	C626891	C6H14O	Sweet, Fruit, Alcohol	0.28±0.01^{a}	0.12±0.01^{c}	0.09±0.00^{d}	0.13±0.01^{bc}	0.11±0.01^{cd}	0.12±0.01^{c}	0.15±0.01^{b}	0.27±0.03^{a}
2-Propanol(M)	C67630	C3H8O	Floral	2.25±1.72^{c}	6.16±1.23^{a}	3.23±0.56^{bc}	2.85±0.43^{c}	3.87±1.33^{bc}	4.64±0.34^{ab}	0.41±0.02^{d}	0.46±0.02^{d}
2-Propanol(D)	C67630	C3H8O	Floral	0.25±0.30^{abc}	0.40±0.26^{a}	0.12±0.04^{abc}	0.08±0.03^{bc}	0.17±0.10^{abc}	0.34±0.10^{abc}	0.03±0.00^{c}	0.05±0.01^{b}
4-Methyl-2-pentanol(M)	C108112	C6H14O	Pungent, Floral, Fruit	9.46±0.21^{d}	12.76±0.45^{b}	8.93±0.18^{d}	7.33±0.18^{e}	15.32±0.43^{a}	12.07±0.60^{c}	0.51±0.04^{f}	0.91±0.11^{f}
4-Methyl-2-pentanol(D)	C108112	C6H14O	Pungent, Floral, Fruit	18.72±1.98^{b}	16.04±0.18^{c}	9.05±0.11^{d}	5.72±0.02^{e}	30.17±0.43^{a}	31.40±0.66^{a}	4.40±0.12^{e}	8.18±0.32^{d}
2-Octanol	C123966	C8H18O	Fresh, Fat, Mushroom	0.18±0.02^{f}	0.57±0.02^{a}	0.23±0.00^{e}	0.33±0.01^{d}	0.37±0.01^{c}	0.13±0.01^{f}	0.26±0.01^{e}	0.48±0.05^{b}
2-Ethyl-1-hexanol	C104767	C8H18O	Green, Rose	0.13±0.02^{d}	0.13±0.01^{d}	0.24±0.01^{b}	0.19±0.01^{c}	0.10±0.01^{e}	0.07±0.00^{f}	0.18±0.00^{c}	0.27±0.01^{a}
1-Propanol	C71238	C3H8O	Alcohol, Candy, Pungent	0.93±0.33^{c}	0.84±0.02^{cd}	0.56±0.01^{de}	0.52±0.02^{e}	0.81±0.03^{cde}	0.69±0.04^{cde}	6.39±0.06^{a}	5.67±0.33^{b}
2-Methyl-2-propanol	C75650	C4H10O	Sweet, Camphor	0.54±0.04^{e}	0.75±0.04^{d}	0.66±0.03^{d}	1.10±0.05^{c}	0.52±0.02^{e}	0.36±0.06^{f}	5.86±0.04^{a}	5.68±0.17^{b}
**Nitriles**											
Hexanenitrile	C628739	C6H11N	Roasted Nut, Bean	0.58±0.02^{a}	0.25±0.00^{d}	0.18±0.02^{e}	0.13±0.00^{f}	0.39±0.02^{c}	0.47±0.02^{b}	0.12±0.01^{f}	0.17±0.03^{e}
Acrylonitrile(D)	C107131	C3H3N	Pungent	1.36±0.32^{c}	1.37±0.02^{c}	1.08±0.02^{c}	1.74±0.08^{c}	1.11±0.05^{c}	1.27±0.39^{c}	9.03±0.12^{b}	10.17±0.95^{a}
Acrylonitrile(M)	C107131	C3H3N	Pungent	0.67±0.10^{c}	0.75±0.01^{c}	0.49±0.01^{c}	0.67±0.08^{c}	0.61±0.01^{c}	0.65±0.18^{c}	7.29±0.13^{b}	18.08±0.32^{a}
**Ketones**											
2-Propanone	C67641	C3H6O	Pungent	1.97±0.19^{c}	2.40±0.20^{b}	1.53±0.08^{de}	2.17±0.05^{bc}	1.71±0.16^{d}	1.68±0.10^{d}	5.15±0.11^{a}	1.29±0.16^{e}
2,3-Pentanedione	C600146	C5H8O2	Caramel, Strawberry, Fruit, Cheese, Brown wine	10.72±0.22^{a}	7.69±0.08^{b}	2.85±0.04^{f}	3.72±0.06^{e}	6.44±0.30^{d}	7.24±0.16^{c}	4.07±0.06^{e}	6.99±0.19^{c}
2-Pentanone(M)	C107879	C5H10O	Fruit, Pungent	2.38±0.12^{c}	2.32±0.11^{c}	7.21±0.10^{b}	8.73±0.23^{a}	2.10±0.09^{d}	1.62±0.07^{e}	0.40±0.02^{g}	0.73±0.09^{f}
2-Pentanone(D)	C107879	C5H10O	Fruit, Pungent	1.03±0.05^{d}	1.40±0.08^{c}	13.88±0.06^{a}	11.34±0.10^{b}	0.67±0.06^{e}	0.30±0.05^{g}	0.42±0.01^{f}	0.72±0.07^{e}
2-Heptanone(M)	C110430	C7H14O	Blue Cheese, Fruit, Green, Nut, Spice	1.02±0.05^{c}	0.77±0.01^{e}	7.73±0.09^{b}	8.07±0.02^{a}	0.70±0.08^{e}	0.98±0.03^{c}	0.71±0.03^{e}	0.87±0.04^{d}
2-Heptanone(D)	C110430	C7H14O	Blue Cheese, Fruit, Green, Nut, Spice	0.27±0.03^{d}	0.26±0.01^{d}	4.52±0.01^{a}	3.41±0.18^{b}	0.19±0.00^{d}	0.18±0.01^{d}	0.29±0.02^{d}	0.50±0.05^{c}
2-Nonanone(M)	C821556	C9H18O	Fragrant, Fruit, Green, Hot Milk	0.16±0.02^{d}	0.13±0.01^{e}	0.59±0.02^{a}	0.59±0.02^{a}	0.08±0.01^{f}	0.12±0.02^{e}	0.22±0.03^{c}	0.34±0.02^{b}
2-Nonanone(D)	C821556	C9H18O	Fragrant, Fruit, Green, Hot Milk	0.04±0.00^{de}	0.03±0.00^{de}	0.15±0.01^{a}	0.12±0.01^{b}	0.03±0.01^{f}	0.03±0.00^{de}	0.04±0.01^{d}	0.07±0.00^{c}
**Aldehydes**											
1-Nonanal(M)	C124196	C9H18O	Fishy, Fat, Floral, Green, Lemon	0.24±0.04^{e}	0.40±0.01^{bc}	0.69±0.01^{a}	0.65±0.04^{a}	0.38±0.02^{c}	0.27±0.02^{de}	0.29±0.01^{d}	0.44±0.02^{b}
1-Nonanal(D)	C124196	C9H18O	Fishy, Fat, Floral, Green, Lemon	0.03±0.00^{d}	0.04±0.01^{c}	0.10±0.00^{a}	0.09±0.01^{a}	0.04±0.00^{c}	0.02±0.00^{d}	0.03±0.01^{cd}	0.06±0.00^{b}
1-Octanal(M)	C124130	C8H16O	Citrus, Fat, Green, Oil, Pungent	0.15±0.01^{f}	0.52±0.03^{d}	0.63±0.01^{b}	0.79±0.02^{a}	0.36±0.01^{e}	0.16±0.01^{f}	0.36±0.00^{e}	0.58±0.06^{c}
1-Octanal(D)	C124130	C8H16O	Citrus, Fat, Green, Oil, Pungent	0.04±0.01^{c}	0.08±0.01^{b}	0.08±0.01^{b}	0.11±0.00^{a}	0.05±0.00^{c}	0.05±0.01^{c}	0.08±0.03^{b}	0.11±0.01^{a}
Heptanal(D)	C111717	C7H14O	Citrus, Fat, Green, Nut	0.04±0.01^{ef}	0.09±0.01^{c}	0.12±0.00^{b}	0.19±0.01^{a}	0.05±0.00^{de}	0.03±0.00^{f}	0.06±0.00^{d}	0.10±0.01^{c}
Heptanal(M)	C111717	C7H14O	Citrus, Fat, Green, Nut	0.13±0.01^{e}	0.61±0.02^{c}	0.84±0.03^{b}	1.15±0.04^{a}	0.37±0.02^{d}	0.15±0.03^{e}	0.39±0.02^{d}	0.57±0.04^{c}
(E)-2-Octenal	C2548870	C8H14O	Dandelion, Fat, Fruit, Grass, Green, Spice	0.07±0.00^{e}	0.17±0.00^{b}	0.14±0.01^{c}	0.22±0.02^{a}	0.15±0.01^{c}	0.05±0.00^{f}	0.10±0.01^{d}	0.15±0.02^{bc}
2-Hexenal	C505577	C6H10O		0.20±0.02^{e}	0.63±0.04^{b}	0.37±0.02^{c}	0.79±0.04^{a}	0.29±0.04^{d}	0.12±0.01^{f}	0.27±0.02^{d}	0.32±0.03^{d}
(E)-2-Heptenal(D)	C18829555	C7H12O	Almond, Fat, Fruit	0.06±0.00^{f}	0.39±0.01^{a}	0.12±0.00^{e}	0.35±0.02^{b}	0.17±0.01^{c}	0.04±0.00^{f}	0.13±0.02^{d}	0.14±0.01^{de}
(E)-2-Heptenal(M)	C18829555	C7H12O	Almond, Fat, Fruit	0.13±0.02^{f}	1.29±0.05^{b}	0.62±0.02^{e}	1.40±0.03^{a}	0.79±0.03^{c}	0.12±0.01^{f}	0.73±0.03^{d}	0.77±0.02^{cd}
Hexanal(D)	C66251	C6H12O	Apple, Fat, Fresh, Green, Oil	0.21±0.00^{f}	2.96±0.07^{c}	2.94±0.02^{c}	4.68±0.10^{a}	1.11±0.04^{e}	0.21±0.02^{f}	4.42±0.07^{b}	2.33±0.04^{d}
Hexanal(M)	C66251	C6H12O	Apple, Fat, Fresh, Green, Oil	2.49±0.15^{f}	10.61±0.27^{c}	8.81±0.03^{d}	12.04±0.02^{b}	6.11±0.13^{e}	2.59±0.19^{f}	13.76±0.03^{a}	13.42±0.71^{a}
(E)-2-Nonenal	C18829566	C9H16O	Paper	0.25±0.04^{e}	0.36±0.02^{cd}	0.28±0.01^{de}	0.39±0.02^{c}	0.25±0.01^{e}	0.21±0.01^{e}	0.56±0.07^{b}	0.78±0.08^{a}
**Pyrazines**											
2-Ethyl-5-methylpyrazine	C13360640	C7H10N2	Fruit, Green	0.19±0.01^{b}	0.18±0.01^{b}	0.08±0.01^{d}	0.15±0.01^{c}	0.09±0.00^{d}	0.09±0.02^{d}	0.62±0.04^{a}	0.09±0.01^{d}
2-Ethyl-3,5-dimethylpyrazine	C13925070	C8H12N2	Broth, Earth, Potato, Roast	0.56±0.02^{b}	0.13±0.01^{d}	0.09±0.00^{de}	0.05±0.00^{e}	0.33±0.06^{c}	0.82±0.04^{a}	0.04±0.01^{e}	0.05±0.01^{e}
**Amines**											
Pyrrolidine	C123751	C4H9N	Pungent	3.58±0.29^{c}	1.17±0.02^{e}	4.56±0.02^{a}	4.04±0.19^{b}	1.00±0.02^{e}	1.46±0.18^{d}	0.31±0.02^{f}	0.49±0.03^{f}
**Alkenes**											
Ethenyl benzene	C100425	C8H8	Paint	0.79±0.02^{d}	0.39±0.02^{f}	1.29±0.04^{a}	1.17±0.03^{b}	0.25±0.03^{g}	0.43±0.01^{f}	0.51±0.05^{e}	0.89±0.01^{c}
**Carboxylic acids**											

2-Methylbutanoic acid	C116530	C5H10O2	Butter, Cheese, Fermented, Sour	3.06±0.24^{a}	0.55±0.03^{c}	0.16±0.01^{d}	0.11±0.00^{d}	0.57±0.01^{c}	0.93±0.12^{b}	0.06±0.00^{d}	0.11±0.00^{d}
3-Methylbutanoic acid	C503742	C5H10O2	Cheese, Pungent	1.92±0.17^{a}	0.73±0.03^{c}	0.25±0.02^{d}	0.17±0.01^{de}	0.81±0.02^{c}	1.04±0.14^{b}	0.07±0.00^{f}	0.12±0.02^{de}
**Sulfur compounds**											
Dipropyl trisulfide(M)	C6028611	C6H14S3	Garlic	7.13±0.11^{a}	5.00±0.16^{c}	1.94±0.11^{e}	1.24±0.06^{g}	4.12±0.04^{d}	5.77±0.30^{b}	0.85±0.04^{h}	1.72±0.17^{f}
Dipropyl trisulfide(D)	C6028611	C6H14S3	Garlic	14.47±0.15^{a}	8.95±0.26^{c}	3.49±0.12^{e}	2.12±0.15^{g}	7.55±0.24^{d}	11.98±0.78^{b}	1.60±0.03^{g}	2.88±0.20^{f}
**Esters**											
Butyl butanoate	C109217	C8H16O2	Floral	0.25±0.02^{e}	0.35±0.02^{d}	0.33±0.02^{d}	0.41±0.04^{c}	0.27±0.01^{e}	0.24±0.01^{e}	0.64±0.03^{b}	0.87±0.02^{a}
Acetic acid ethyl ester(M)	C141786	C4H8O2	Aromatic, Brandy, Grape	2.90±0.16^{d}	6.25±0.09^{a}	4.40±0.10^{c}	5.63±0.25^{b}	4.54±0.07^{c}	3.07±0.09^{d}	1.22±0.04^{e}	0.26±0.01^{f}
Acetic acid ethyl ester(D)	C141786	C4H8O2	Aromatic, Brandy, Grape	0.69±0.02^{b}	0.42±0.01^{e}	0.93±0.02^{a}	0.62±0.04^{c}	0.40±0.02^{e}	0.49±0.04^{d}	0.25±0.02^{f}	0.10±0.01^{g}
Hexyl 2-methyl butyrate	C10032152	C11H22O2	Strawberry	0.16±0.01^{c}	0.17±0.01^{c}	0.20±0.01^{b}	0.21±0.01^{b}	0.15±0.01^{c}	0.11±0.01^{d}	0.20±0.01^{b}	0.32±0.03^{a}
Ethyl 3-hydroxybutanoate	C5405414	C6H12O3	Marshmallow, Roasted Nut	0.04±0.00^{de}	0.06±0.00^{c}	0.03±0.00^{e}	0.05±0.01^{cd}	0.04±0.01^{cde}	0.03±0.00^{e}	0.34±0.01^{a}	0.07±0.01^{b}
Butyl propanoate	C590012	C7H14O2	Red Fruit, Strawberry	3.68±0.10^{b}	1.61±0.01^{e}	2.62±0.02^{d}	1.68±0.04^{e}	3.39±0.10^{c}	4.32±0.03^{a}	0.23±0.04^{g}	0.35±0.03^{f}
Methyl acetate(D)	C79209	C3H6O2	Ester, Green	0.41±0.02^{c}	0.54±0.01^{c}	0.39±0.04^{c}	0.41±0.02^{c}	0.34±0.01^{c}	0.26±0.04^{c}	23.02±0.49^{a}	4.08±0.33^{b}
Methyl acetate(M)	C79209	C3H6O2	Ester, Green	0.13±0.01^{c}	0.12±0.00^{cd}	0.09±0.00^{de}	0.13±0.01^{cd}	0.09±0.01^{de}	0.07±0.00^{e}	2.86±0.03^{b}	5.80±0.05^{a}

Results are means ±standard deviation (n=3). Values in the same row with different letters are significantly different ($p< 0.05$). The flavor descriptions are sourced from https://www.femaflavor.org/flavor-library, https://www.flavornet.org/, and https://www.odour.org.uk/information.html.
